# CRISPR/Cas9-mediated abrogation of CD95L/CD95 signaling-induced glioma cell growth and immunosuppression increases survival in murine glioma models

**DOI:** 10.1007/s11060-022-04137-x

**Published:** 2022-11-10

**Authors:** Clara Quijano-Rubio, Manuela Silginer, Michael Weller

**Affiliations:** 1 Laboratory of Molecular Neuro-Oncology, Department of Neurology, University Hospital Zurich, Zurich, Switzerland; 2 University of Zurich, Zurich, Switzerland

**Keywords:** CD95, CD95L, CRISPR/Cas9, Glioblastoma, Immunosuppression

## Abstract

**Purpose:**

Glioblastoma is the most common brain tumor in adults and is virtually incurable. Therefore, new therapeutic strategies are urgently needed. Over the last decade, multiple growth-promoting functions have been attributed to CD95, a prototypic death receptor well characterized as an apoptosis mediator upon CD95L engagement. Strategic targeting of non-apoptotic or apoptotic CD95 signaling may hold anti-glioblastoma potential. Due to its antithetic nature, understanding the constitutive role of CD95 signaling in glioblastoma is indispensable.

**Methods:**

We abrogated constitutive *Cd95* and *Cd95l* gene expression by CRISPR/Cas9 in murine glioma models and characterized the consequences of gene deletion in vitro and in vivo.

**Results:**

Expression of canonical CD95 but not CD95L was identified in mouse glioma cells in vitro. Instead, a soluble isoform-encoding non-canonical *Cd95l* transcript variant was detected. In vivo, an upregulation of the membrane-bound canonical CD95L form was revealed. *Cd95* or *Cd95l* gene deletion decreased cell growth in vitro. The growth-supporting role of constitutive CD95 signaling was validated by *Cd95* re-transfection, which rescued growth. In vivo, *Cd95* or *Cd95l* gene deletion prolonged survival involving tumor-intrinsic and immunological mechanisms in the SMA-497 model. In the GL-261 model, that expresses no CD95, only CD95L gene deletion prolonged survival, involving a tumor-intrinsic mechanism.

**Conclusion:**

Non-canonical CD95L/CD95 interactions are growth-promoting in murine glioma models, and glioma growth and immunosuppression may be simultaneously counteracted by *Cd95l* gene silencing.

**Supplementary Information:**

The online version contains supplementary material available at 10.1007/s11060-022-04137-x.

## Introduction

CD95, also referred to as Fas or APO-1, is a pleiotropic cytokine receptor that, upon cognate stimulation with CD95 ligand (CD95L), may lead either to tumor-suppressing or tumor-promoting signaling. Initially, CD95L/CD95 interactions were described to result in the apoptotic cell death of CD95-expressing cells. When induced in cancer cells, CD95 signaling was therefore considered tumor-suppressing. Accordingly, agonistic CD95 targeting to induce apoptosis in CD95-expressing cancer cells was investigated upon the discovery of CD95 [1, 2], including in glioblastoma [3, 4]. However, therapeutic stimulation of apoptotic CD95 signaling in cancer cells is generally considered clinically impracticable due to major associated side effects [5, 6]. Furthermore, cumulative evidence suggests that cognate interactions between CD95L and CD95 may likewise prompt non-apoptotic tumor-promoting features such as proliferation, invasiveness and stemness in CD95-expressing cancer cells [7–12] in various malignancies including glioblastoma [13–15]. The notion of such tumor-promoting CD95 signaling entailed a paradigm shift in therapeutic CD95 targeting. Specifically, CD95 signaling inhibition using CD95L scavenging strategies rather than stimulation was considered for the treatment of glioblastoma [16].

Therapeutic CD95L inhibition has been generally oriented at blocking non-apoptotic, tumor-promoting signal transduction in cancer cells [17, 18]. However, there is also a scenario where apoptotic CD95 signaling may be tumorigenic, that is, when the apoptotic cell is not a cancer cell but a CD95-expressing effector immune cell. CD95 expression in immune cells renders them susceptible to apoptosis induced by CD95L expressed in the tumor. Interactions between CD95L expressed by glioma cells [19–21] or the tumor microenvironment with CD95 expressed in leukocytes have been proposed to induce immune cell apoptosis and subsequent immunosuppression [22–25]. However, this notion remains controversial since T cell killing assays have failed to reproduce this tumor counterattack [26], and studies where tumor counterattack was shown in vitro but not in vivo have been published, too [27].

Nevertheless, strategic inhibition of CD95 signaling to simultaneously block CD95-mediated cancer cell growth and the apoptotic death of cells in the tumor microenvironment with antitumor potential, including CD95-expressing immune effector cells, may represent a suitable alternative therapeutic strategy targeting the CD95-CD95L system. In glioblastoma, a virtually incurable cancer to date, such strategies may be of high interest since they may theoretically affect two of the major factors contributing to glioblastoma aggressiveness and resistance to therapy: the rapid and infiltrative glioblastoma cell growth and the highly immunosuppressive tumor milieu. Therefore, in this study, we explored comprehensively the role of cancer cell-intrinsic and tumor microenvironment-mediated CD95L/CD95 interactions using syngeneic mouse glioma models. To ensure the study of constitutive CD95 signaling without artifacts, specific gene deletion of *Cd95* and *Cd95l* was utilized here to elucidate the role of CD95 signaling in glioblastoma models.

## Material and methods

### Cell lines

SMA-497, SMA-540 and SMA-560 cells were kindly provided by Dr. D. D. Bigner (Durham, NC) and GL-261 cells were obtained from ATCC (Rockville, MD). Human embryonic kidney (HEK) 293T cells were purchased from Open BioSystems (Huntsville, AL). Cells were grown as adherent monolayers in Dulbecco´s modified Eagle´s medium (DMEM) supplemented with 10% fetal calf serum (FCS) and 2 mM L-glutamine (Gibco, Waltham, MA). Cells were regularly tested for mycoplasma contamination using the enzymatic MycoAlert^™^ PLUS Mycoplasma Detection Kit (Lonza, Basel, Switzerland).

### Animal studies

Details for animal study methods are provided in Supplementary Material and Methods. Briefly, 500 − 100’000 glioma cells were stereotactically implanted into the right striatum of 4 to 16 weeks old mice. The onset of neurological symptoms defined end-stage survival.

### Statistical analysis

Data are expressed as mean and standard deviation (SD) or standard error of the mean (SEM) and are representative of several independent experiments. Statistical analyses were performed with GraphPad Prism version 8 (GraphPad Software, San Diego, CA, http://www.graphpad.com). One- or two-way ANOVA with Bonferroni post-hoc tests were applied for multiple comparison significance assessments. Survival curve estimation was performed by Kaplan-Meier survival analysis and statistical significance in survival experiments was assessed by means of log-rank test. Significance levels for all analyses are *p < 0.05; **p < 0.01; ***p < 0.001; ****p < 0.0001.

## Results

### CD95 and CD95L expression in murine glioma cell lines

**Fig. 1:**
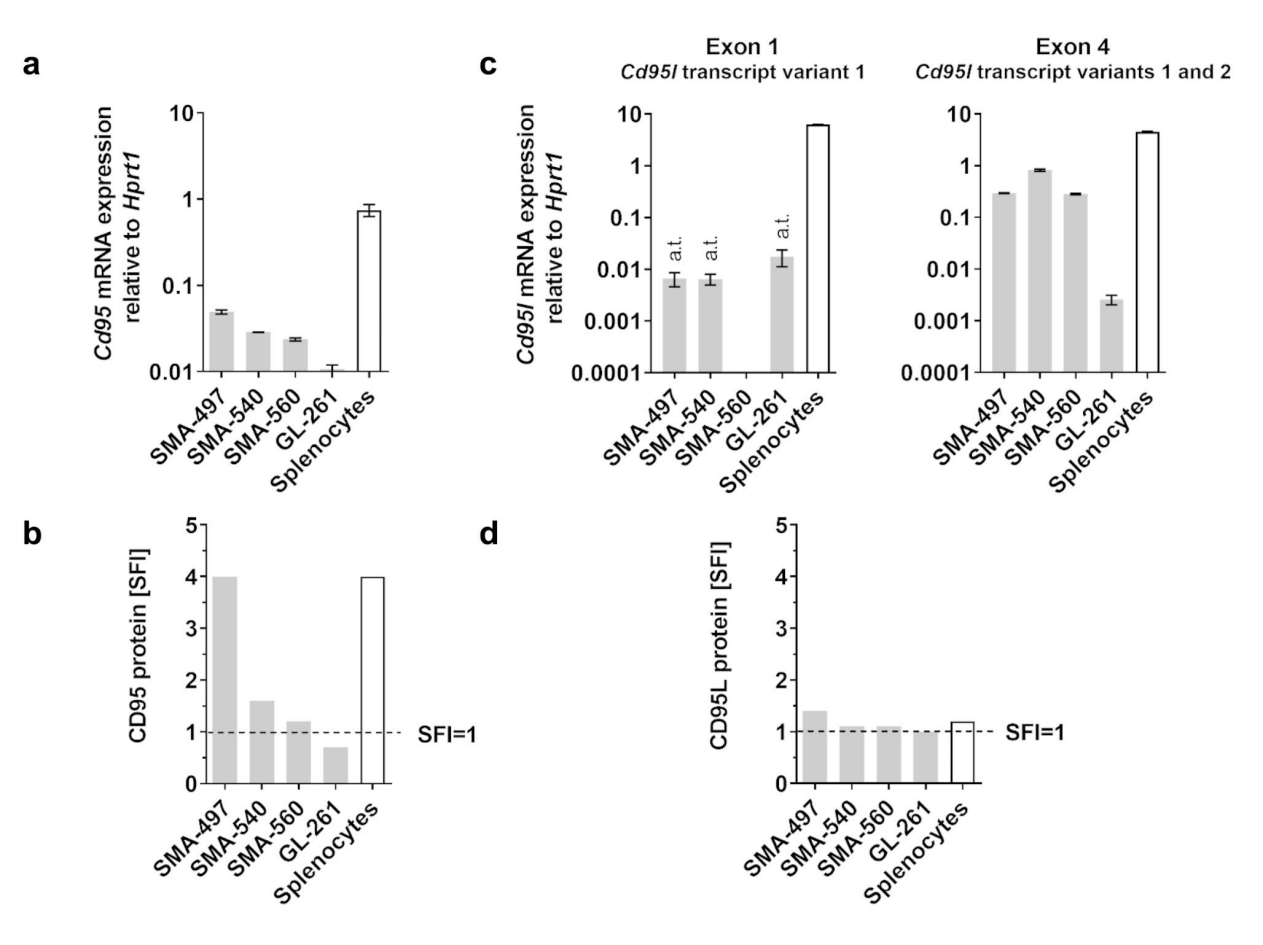
CD95 and CD95L expression in mouse glioma cells. a. SMA-497, SMA-540, SMA-560 or GL-261 cells were assessed for expression of *Cd95* by PCR using *Hprt1* as endogenous control. b. Protein levels were assessed by flow cytometry. c. To assess *Cd95l* mRNA expression, two primer pairs, one targeting the first exon of the complete *Cd95l* coding sequence and one targeting the fourth exon of the complete *Cd95l* coding sequence, were used. d. CD95L protein levels were assessed by flow cytometry. Activated splenocytes (2 µg/ml concanavalin A) were included as positive control for CD95 protein and *Cd95* mRNA and for *CD95l* mRNA. Data in a,c are presented as mean and SD. In c, a.t. indicates C_T_ values above reliability threshold, C_T_>32.

*Cd95* mRNA expression was confirmed in four murine glioma cell lines (SMA-497, SMA-540, SMA-560, GL-261) (Fig. 1a). CD95 protein was detected on the surface of SMA-497 and SMA-540, but only at low levels (SFI < 1.5) in SMA-560 and not at all in GL-261 cells (Fig. 1b, S1a) (Note S1). Two murine CD95L protein isoforms have been described: the full-length canonical isoform, containing an extracellular domain, a transmembrane domain and a cytoplasmic domain; and a shorter non-canonical isoform, CD95Ls, which lacks the cytoplasmic and the transmembrane domains [28]. Full-length CD95L is encoded by the *Cd95l* transcript variant 1, consisting of four exons, and CD95Ls is encoded by the *Cd95l* transcript variant 2, consisting of part of the first and the fourth exons of the canonical *Cd95l* sequence [29]. Canonical *Cd95l* was expressed in none of the glioma cell lines in vitro, as assessed by RT-qPCR using a primer pair targeting a region of the first *Cd95l* exon uniquely present in transcript variant 1 (Fig. 1c, left). However, *Cd95l* transcript expression was detected in all cell lines using primers targeting a region of the fourth exon of *Cd95l*, common to transcripts 1 and 2 (Fig. 1c, right). Since *Cd95l* transcript was detected using primers targeting both variants but not with primers targeting variant 1 exclusively, we inferred that murine glioma cells express only the transcript variant 2, encoding the soluble CD95L isoform CD95Ls. Accordingly, CD95L protein was not detected on the surface of either glioma cell line, but also not on splenocytes (Fig. 1d, S1b) (Note S2). Exposure to temozolomide has been suggested to upregulate CD95L in human glioblastoma cells [13]. Cell surface CD95L expression was, however, not induced by exposure to temozolomide at 10 µM or 100 µM for 24 to 120 h in SMA-497 cells in vitro (Fig. S2).

#### CD95L is upregulated in murine gliomas in vivo

**Fig. 2:**
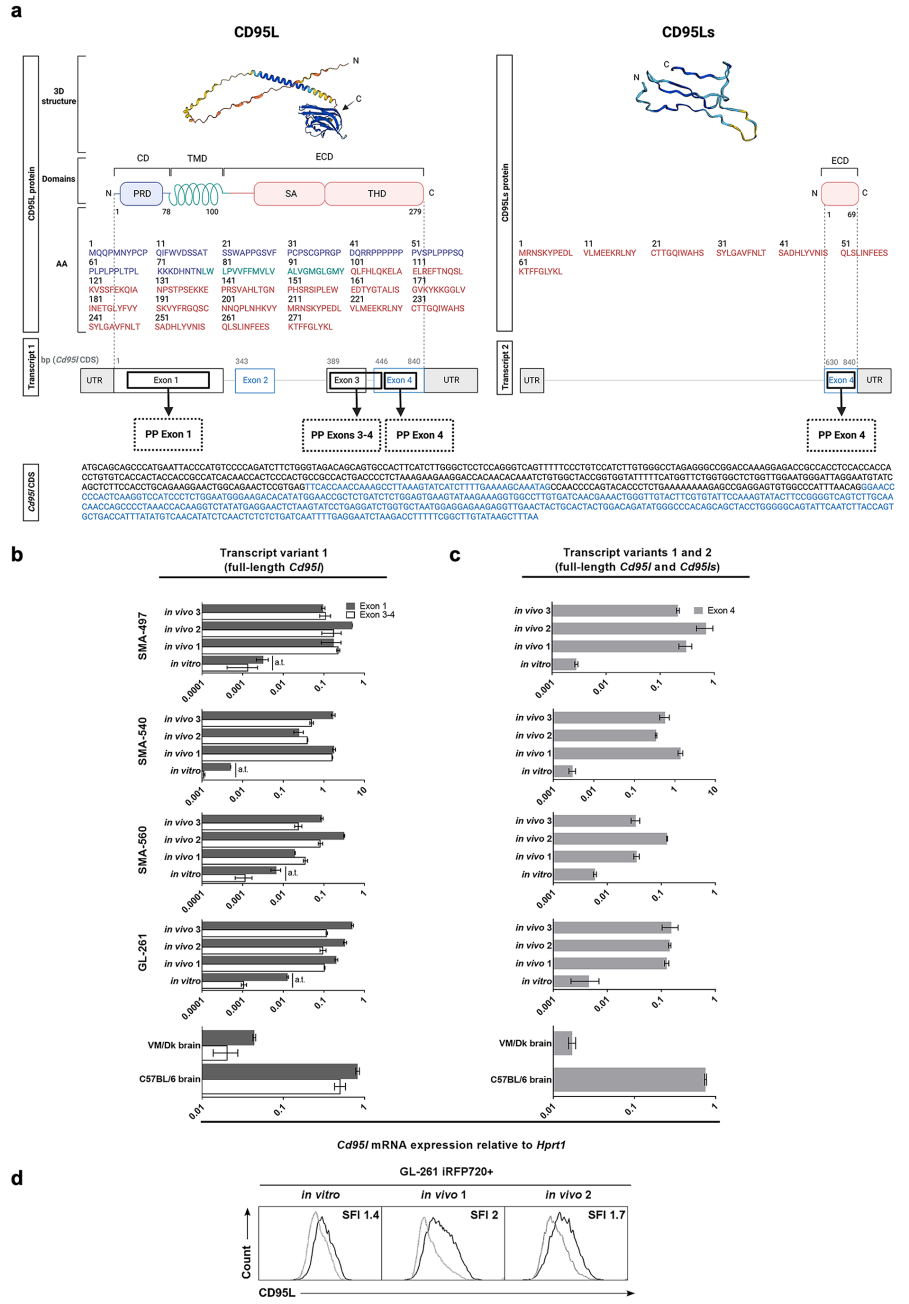
CD95L expression in murine gliomas *in vivo*. a. Diagram of the two known murine *Cd95l* transcript variants and their sequence correspondence with the canonical *Cd95l* coding sequence (*Cd95l* CDS) and the putative murine CD95L and CD95Ls protein domains (created with BioRender.com). Three primer pairs (PP) used for the analysis of *Cd95l* transcript expression in vivo and their approximate amplifying regions in the transcript variants they are predicted to amplify are depicted. The CDS sequence, transcript elements, protein sequences and domain correspondence depiction were based on the data annotated in the Ensembl and UniProt databases [29, 35]. The 3D protein structures are AlfaFold predictions. The CD95L structure was extracted from the AlphaFold database [36] and the CD95Ls 3D structure was generated using the AlphaFold Colab notebook [37]. Structure coloring indicates model confidence (dark blue, pLDDT > 90; light blue, pLDDT 90 − 70; yellow, pLDDT 70 − 50; orange, pLDDT < 50). CD, death domain; TMD, transmembrane domain; ECD, extracellular domain; PRD, proline-rich domain; SA, self-assembly domain; THD, TNF homology domain. b,c. Cells were maintained in vitro or implanted in syngeneic mice and tumors from end-stage glioma-bearing mice were isolated. Expression of *Cd95l* mRNA in tumors and the surrounding tumor-bearing brain tissue (C57BL/6 brain and VM/Dk brain) was analyzed by RT-qPCR using primer pairs targeting the first exon of *Cd95l* or the sequence spanning the third and four exons of the complete *Cd95l* coding sequence (b) or a primer pair targeting the fourth exon of *Cd95l* (c). *Hprt1* was used as internal control. Three tumor-bearing mice (in vivo 1, in vivo 2, in vivo 3) per model were studied. Data are represented as mean and SD (a.t., C_T_ values above reliability threshold, C_T_>32). d. iRFP720-labelled GL-261 cells (GL-261 iRFP720+) were implanted in syngeneic mice. Tumors from end-stage mice were isolated and analyzed by flow cytometry. Flow cytometry histograms showing cell surface CD95L protein levels in GL-261 cells gated based on iRFP720 positivity are depicted. SFI, specific fluorescence index (median fluorescence intensity of anti-CD95L antibody, black ÷ median fluorescence intensity of isotype control, grey).

The analysis of *Cd95l* transcript levels in the tumors of SMA-497, SMA-540, SMA-560 and GL-261 glioma cell-bearing mice revealed an upregulation of canonical *Cd95l* mRNA in vivo (Fig. 2b,c). *Cd95l* transcript levels were studied using primers targeting part of the first *Cd95l* exon or the third-to-fourth exon junction, exclusive of the canonical transcript variant 1 (Fig. 2a,b) and primers targeting part of the fourth *Cd95l* exon common to both the canonical transcript variant 1 and the CD95Ls-encoding variant 2 (Fig. 2a,c). CD95L was also detected on the surface of iRFP720-labelled GL-261 cells isolated from end-stage tumor-bearing mice, confirming not only protein expression but also glioma cell origin (Fig. 2d).

### CD95 and CD95L gene disruption in mouse glioma cells

To investigate the role of constitutive CD95 signaling in murine glioma cells, CD95 or CD95L were knocked out by means of CRISPR/Cas9. CD95 depletion was mediated by two sgRNA directing the disruption of the second *Cd95* exon, which is shared by all *Cd95* transcript variants and encodes the N-terminus region of the extracellular CD95 domain, essential for CD95L binding [30]. CD95L depletion was mediated by a sgRNA directing a double-strand DNA break on the first *Cd95l* exon, which encodes the transmembrane CD95L domain, only present in canonical CD95L; and a sgRNA directing a double-strand DNA break in a region of the fourth *Cd95l* exon, which encodes part of the extracellular CD95L domain, common to both CD95L isoforms.

**Fig. 3:**
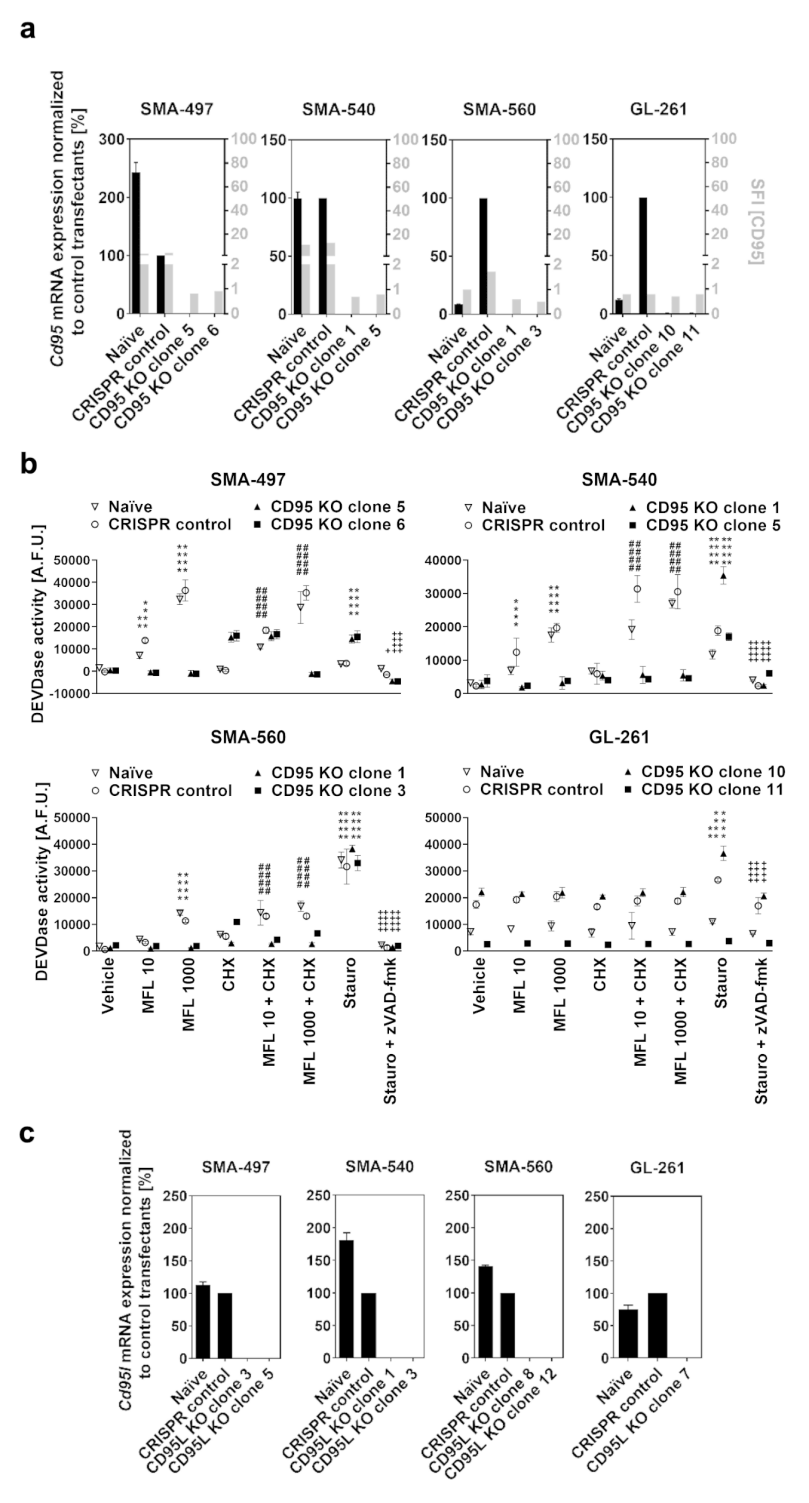
CD95 and CD95L CRISPR knockout (KO) in mouse glioma cells. *Cd95* or *Cd95l* genes were knocked out via CRISPR/Cas9. a. Clonal CD95 KO cells were assessed for expression of the *Cd95* transcript sequence spanning two predicted double-strand DNA break sites by RT-qPCR using *Hprt1* as endogenous control and of protein by cell surface flow cytometry. RT-qPCR data are expressed as mean and SD. SFI, specific fluorescence index (median fluorescence intensity of experimental antibody ÷ median fluorescence intensity of isotype control). b. Naïve, CRISPR control or CD95 KO cells were stimulated with 10 or 1000 ng/ml exogenous CD95L (Mega-Fas-Ligand, MFL) in the absence or presence of cycloheximide (CHX, 10 µg/ml) for 6 h and assessed for Ac-DEVD-amc-cleaving (DEVDase) activity. As a positive control, cells were treated with 1 µM staurosporine (stauro). As a negative control, cells were treated with 10 µM zVAD-fmk in combination with staurosporine. A representative independent experiment is shown for each cell line. Data are expressed as mean and SD of six replicates. Statistical significances were assessed by two-way ANOVA followed by Bonferroni’s post hoc test (**p < 0.01, ****p < 0.0001 versus vehicle; ####p < 0.0001 versus CHX; +p < 0.05, ++++p < 0.0001 versus stauro). A.F.U., arbitrary fluorescence units. c. Clonal CD95L KO cells were assessed for expression of the *Cd95l* transcript sequence targeted by a sgRNA directing a Cas9-mediated double-strand DNA break in the fourth exon of *Cd95l* by RT-qPCR using *Hprt1* as endogenous control. Data are expressed as mean and SD. Naïve refers to non-transfected cells, and CRISPR control to cells transfected with non-targeting sgRNA/*pSpCas9*(BB)-2 A-*GFP* plasmids.

CD95 knockout clonal sublines were selected based on the absence of the mRNA sequence situated between the two predicted sgRNA-directed double-strand DNA break sites (Fig. 3a; left y axis). CD95 knockout was confirmed on protein level based on the absence of cell surface protein (Fig. 3a; right y axis, S3). Because of the lack of cell surface CD95 detection in naïve SMA-560 and GL-261 cells, CD95 knockout confirmation in these cell lines relied uniquely on the absence of the target mRNA sequence.

SMA-497, SMA-540 and SMA-560 CD95 knockout sublines, contrary to naïve and CRISPR control cells, did not respond to stimulation with exogenous CD95L by increasing DEVD-amc cleaving activity, which characterizes the activity of the apoptotic effector caspase 3 as part of canonical CD95 signaling. In these sublines, DEVD-amc cleaving activity was also abrogated during stimulation with CD95L in combination with the protein synthesis inhibitor cycloheximide, which sensitizes glioma cells to CD95-mediated apoptosis induction [4]. The increase of DEVD-amc cleaving activity upon CD95L stimulation in SMA-560 naïve and control cells and its abrogation upon CD95 knockout suggested the expression of functional CD95, although undetectable by flow cytometry. An increase in the DEVD-amc cleaving activity upon stimulation with CD95L was not observed in GL-261 cells, indicating either insufficient CD95 protein levels or resistance to CD95-mediated apoptosis (Fig. 3b). CD95L knockout clonal sublines were selected based on the absence of the *Cd95l* mRNA target sequence of the sgRNA targeting exon 4 (Fig. 3c).

### Phenotypic characterization of CD95 and CD95L knockout in murine gliomas in vitro

**Fig. 4:**
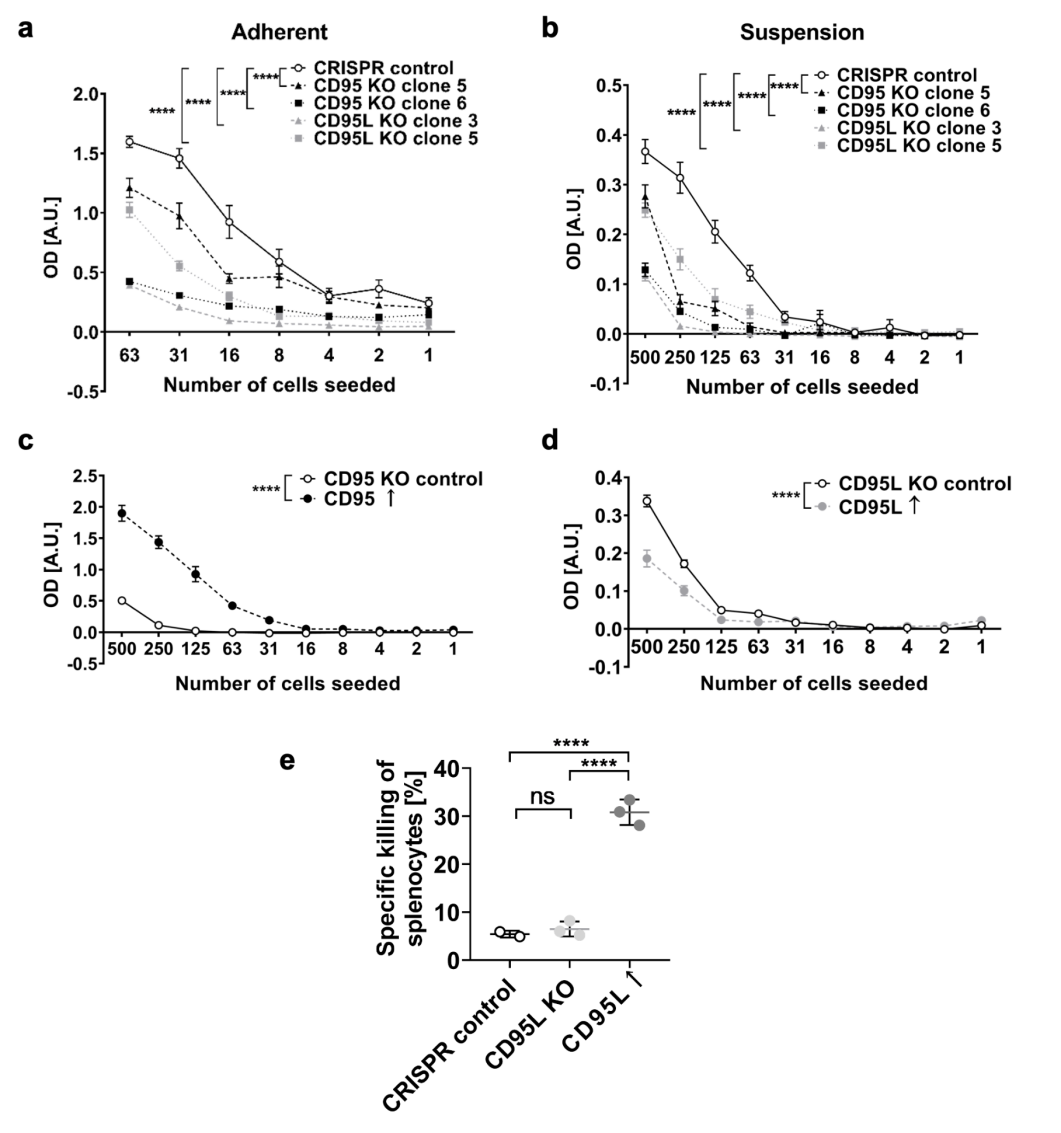
Effect of CD95 and CD95L knockout (KO) in SMA-497 cell growth and T cell killing *in vitro*. a,b. The growth of CRISPR control, CD95 KO or CD95L KO SMA-497 cells was estimated by crystal violet staining in limiting dilution assays of cells grown in adherence conditions in the presence of fetal calf serum (a) or by MTT assay in cells cultured as spheres in suspension in the absence of fetal calf serum, but with EGF and FGF supplementation (b). c,d. Cell growth of adherent CD95 KO (clone 6) or CD95L KO (clone 3) SMA-497 cells transfected with *Cd95* or *Cd95l*, respectively, was estimated in limiting dilution assays by crystal violet staining. Data in a-d are expressed as mean and SEM of representative experiments. Statistical significances (main column effect) were determined by means of a two-way ANOVA test followed by Bonferroni’s post hoc test. Data were reproduced in three independent experiments. e. Specific killing of activated splenocytes after 24 h co-culture with CRISPR control, CD95L KO (clone 3) or *Cd95l*-transfected CD95L KO (clone 3) SMA-497 cells. Data in e are expressed as mean and SD. Statistical significances were calculated by one-way ANOVA followed by Bonferroni’s post hoc test. A.U., arbitrate units; ns, not significant; ****, p < 0.0001.

CD95 and CD95L knockout SMA-497 sublines exhibited reduced cell growth under conventional adherent culture conditions (Fig. 4a). Under suspension spheroid stem cell culture conditions, the growth reduction upon CD95 knockout was accentuated (Fig. 4b). The cell growth decrease upon knockout was reproduced in CD95L knockout SMA-540, SMA-560 and GL-261 cells (Fig. S4a-c) but not in CD95 knockout SMA-540, SMA-560 and GL-261 sublines, which exhibited major clonal variation (data not shown). To confirm that the observed cell growth reduction in various CD95 knockout clones was a specific consequence of CD95 depletion, CD95 knockout SMA-497 cells were re-transfected with *Cd95*. Indeed, cell growth was rescued in CD95 knockout cells re-expressing CD95 (Fig. 4c, S5). However, CD95 overexpression in non-transfected, constitutively CD95 and CD95L-expressing naïve and CRISPR control cells did not promote cell growth (Fig. S6), suggesting that CD95 signaling stably maintains cell growth, but does not accelerate growth with a linear dose response relationship.

### Functional difference of CD95Ls and canonical full-length CD95L

Transfection of CD95L knockout cells with the complete coding sequence of *Cd95l* resulted in reduced cell growth (Fig. 4d) likely due to augmented CD95L-induced apoptosis, since growth reduction was not observed upon transfection of *Cd95l* into CD95 knockout cells (data not shown). Furthermore, contrary to naïve murine glioma cells, which have been observed to exclusively express the CD95Ls-encoding *Cd95l* transcript in vitro, canonical *Cd95l* transfectants were capable of killing activated splenocytes, presumably via CD95L-CD95-mediated apoptosis (Fig. 4e). A decrease in specific killing of splenocytes was observed upon CD95L knockout neither in SMA-497 nor in SMA-540, SMA-560 or GL-261 cells (Fig. 4e, data not shown).

Despite evident mRNA expression upon *Cd95l* transfection (Fig. S5c, S7a), cell surface CD95L was not observed (Fig. S5d, S7b). However, CD95L was detected in the lysates and supernatants of *Cd95l* transfectants, using an antibody with affinity for the canonical CD95L extracellular domain (Fig. S8). In cell lysates, a ~40 kDa band, corresponding to the CD95L predicted mass, was observed while in cell culture supernatants a band corresponding to a ~25 kDa protein was additionally detected. Since the expected mass of CD95Ls is ~8–16 kDa [31], this band likely resulted from degradation and cleavage upon the artificial condition of overexpression (Note S3).

### *Cd95* or *Cd95l* gene deletion delay growth in syngeneic murine glioma models

**Fig. 5:**
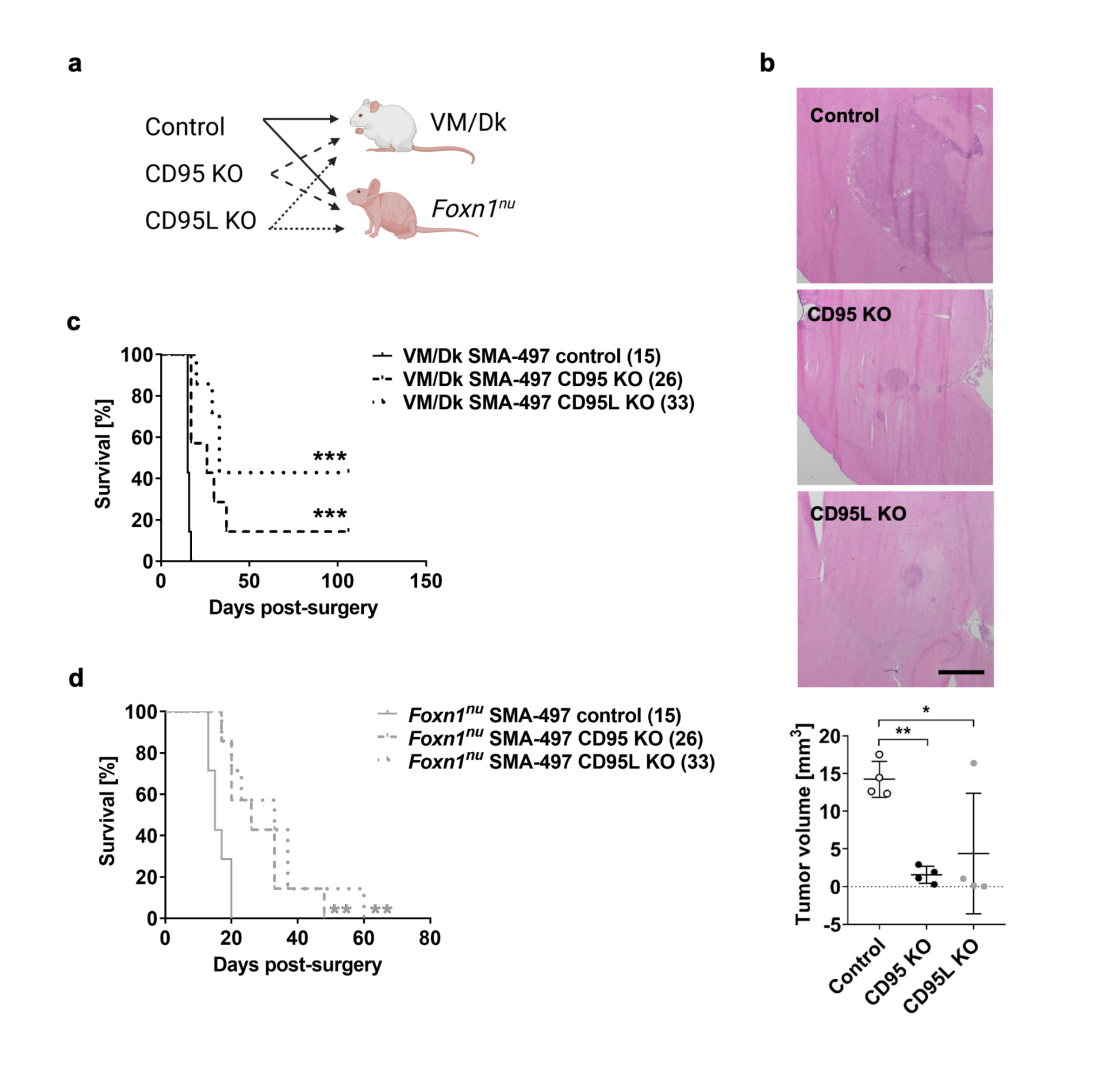
Effect of CD95 and CD95L knockout (KO) in the SMA-497 model in vivo. a. Syngeneic (immunocompetent) VM/Dk or athymic (immunocompromised) *Foxn1*^*nu*^ mice were orthotopically implanted with control (naïve and CRISPR control), CD95 KO (clones 5 and 6) or CD95L KO (clones 3 and 5) SMA-497 cells (illustration created with BioRender.com). b. Tumor volumes from animals sacrificed at the first onset of symptoms were determined by hematoxylin/eosin staining (scale bar = 1 mm). Data are expressed as mean and SD of n = 4 VM/Dk mice. Statistical significances between CD95 KO or CD95L KO and control tumor-bearing brains were determined by means of a one-way ANOVA test followed by Bonferroni’s post hoc test. c,d. End-stage survival was recorded. Statistical significances between CD95 KO or CD95L KO glioma-bearing mice and control glioma-bearing mice within the same mouse strain were determined by means of a log-rank test. Median survival in days provided in brackets (n = 7 mice per group). *, p < 0.05; **, p < 0.01; ***, p < 0.001.

Syngeneic VM/Dk mice orthotopically implanted with CD95 or CD95L knockout SMA-497 cells (Fig. 5a) developed smaller tumors than mice implanted with control cells (Fig. 5b). Control tumors appeared to exhibit less regular borders than CD95L knockout tumors (Fig. 5b, S9a). However, significant differences in the number of tumor satellites, quantified to infer invasion, were not revealed between groups, despite a trend towards reduced satellite number in CD95L knockout tumors potentially driven by tumor size differences (Fig. S9a). Immunocompetent syngeneic mice implanted with CD95 or CD95L knockout SMA-497 cells survived significantly longer than mice implanted with control cells (Fig. 5c). The median survivals of control, CD95 knockout and CD95L knockout tumor-bearing mice were 15, 26 and 33 days, respectively.

### Cancer cell-intrinsic and immunological mechanisms mediate increased survival upon CD95L knockout in the SMA-497 model

The increase in median survival observed in fully immunocompetent mice implanted with CD95 or CD95L knockout cells was reproduced in mature T cell-deficient *Foxn1*^*nu*^ mice (Fig. 5d). However, we noted differences by mouse strain for overall outcome: while 40% of immunocompetent mice bearing CD95L knockout tumors achieved long-term survival (Fig. 5c) and did not develop neurologic symptoms after tumor rechallenge (data not shown), none of the CD95L knockout cell-bearing immunodeficient mice survived for more than 60 days after glioma cell implantation (Fig. 5d). These data suggest a glioma CD95L-mediated T cell suppression that is abrogated in CD95L-depleted glioma cells.

Mean CD3^+^ T cell numbers in the tumors at an early time point did not differ overall between groups. However, while only 50% of CD95L-expressing control or CD95 knockout tumors exhibited CD3^+^ T cell infiltration, CD3^+^ T cells were observed in 100% of CD95L knockout tumors (Fig. S9b). Significant inter-group differences in the numbers of CD11b^+^ microglia/macrophages and CD31^+^ endothelial cells were not revealed either (Fig. S9c,d) (Note S4).

Conversely, in syngeneic GL-261 gliomas, tumor intrinsic effects only mediated increased survival upon CD95L knockout (Fig. S10a) and immune cell-dependency was not observed (Fig. S10b-d) (Note S5).

## Discussion

The role of CD95 signaling in glioblastoma and in many other cancers has remained obscure and controversial, rendering efforts at targeting these molecules therapeutically challenging. Careful consideration needs to be given to the fact that CD95L/CD95 interactions are pleiotropic and subsequent signaling may entail a complex network of not yet completely elucidated signaling pathways leading to distinct outcomes, contributing to either tumor suppression or progression in a context-dependent manner.

Here we sought to gain insight into the function of CD95 and CD95L in murine glioma models using CRISPR/Cas9-mediated gene deletion as the key experimental strategy. We demonstrate that the spontaneous (SMA-497, SMA-540, SMA-560) and chemically induced (GL-261) murine glioma models express canonical *Cd95* and a non-canonical soluble *Cd95l* splice variant, encoding a soluble isoform of CD95L (CD95Ls) in vitro (Fig. 1). Full-length canonical *Cd95l* is expressed only when glioma cells are implanted in syngeneic mouse brains (Fig. 2). To disrupt CD95/CD95L interactions, we introduced genetic deletions in *Cd95* and *Cd95l* by CRISPR/Cas9-mediated knockout.

A growth reduction was evidenced upon CD95 knockout (Fig. 4), although major clonal variation was observed. CD95 KO clones showing no phenotype may have undergone genetic compensation, a phenomenon suggested in various studies involving genetic editing [32]. In fact, we showed a growth rescue upon *Cd95* retransfection, confirming the specificity of CD95 deletion in the growth reduction observed in the CD95 knockout sublines that exhibited a phenotype (Fig. 4). Unlike CD95 re-expression in CD95 knockout cells, CD95 overexpression in parental cells did not increase growth, suggesting that a threshold level of CD95 maintains glioma cell growth whereas supraphysiological levels exert no additional biological effect.

All glioma cell lines were sensitive to CD95-mediated apoptosis (Fig. 3). Therefore, in contrast to previous reports which suggested resistance to CD95-mediated apoptosis as a requisite for growth-promoting CD95 signaling [7, 14], we report growth-promoting constitutive CD95 signaling in CD95-mediated apoptosis-sensitive glioma cells.

Despite the impossibility of detecting CD95Ls protein due to the lack of isoform-specific antibodies, all CD95L knockout cells exhibited a marked growth reduction in vitro (Fig. 4, S4). At the time of its discovery, CD95Ls was described to negatively regulate apoptosis [31]. Our data raise the possibility that CD95Ls may, in fact, induce non-apoptotic tumor-promoting signaling.

In vivo, knockout of CD95 or CD95L in the SMA-497 and GL-261 models had both shared and model-specific effects: *Cd95l* gene depletion uniformly prolonged survival in both models, whereas *Cd95* deletion prolonged survival in the SMA-497 model only (Fig. 5, S10). This may be explained by the low-to-null constitutive level of CD95 in GL-261 cells. Although tumor invasiveness has been reported to be inhibited in the murine SMA-560 glioma upon pharmacologic CD95L blockade [14, 17], significant differences in the number of tumor satellites were not observed in our SMA-497 model. Tumors formed by CD95 and CD95L knockout SMA-497 cells were significantly smaller than control tumors, suggesting that the decreased tumorigenicity of CD95 and CD95L knockout cells is mainly driven by growth inhibition. Reduced tumor incidence and size have also been described upon CD95 depletion in ovarian and liver cancer [12].

While a similar median survival gain was observed in mature T cell-competent and deficient mice, long-term surviving mice after inoculation of CD95L knockout SMA-497 cells were exclusively observed in immunocompetent mice. Furthermore, CD3^+^ T cells infiltrated all CD95L KO tumors but not all CD95L-expressing tumors (Fig. S9), suggesting that CD95L halts CD3^+^ T cells in some mice, presumably due to apoptosis induction. Therefore, we propose that the abrogation of tumor CD95L acting on CD95 expressed on immune cells may allow immunocompetent mice to achieve long-term survival more frequently. Of note, recent observations of CD95-dependent natural killer cell suppression in triple negative breast cancer models [33, 34] indicate that the growth delay of CD95-depleted tumors may not only depend on slowed growth properties as documented in vitro (Fig. 4), but also on altered natural killer cell activity, which is retained in the immunocompromised mouse models used in the present study. Overall, based on our results, we hypothesize that cancer cell-intrinsic growth-promoting CD95 signaling may be mediated by CD95Ls while the potential CD95L-mediated T cell killing is likely induced by canonical CD95L. This is because we did not observe apoptosis-inducing capacity in cells expressing only CD95Ls, but only growth inhibition upon CD95L knockout (Fig. 4a,b, S4). In contrast, ectopic expression of full length CD95L, which is expressed in vivo but not in vitro (Fig. 2), resulted in specific splenocyte killing (Fig. S8). The survival gain mediated by *Cd95l* gene deletion was maintained in GL-261 cell-bearing *Rag*^*-/-*^ mice lacking T and B cells as well as in *Fas*^*lpr*^ mice lacking functional CD95, suggesting a tumor-intrinsic effect of CD95L disruption only that might involve interactions with other death ligands or even backward signaling.

Limitations of our study include the risk of confounding factors derived from the use of clonal sublines and potential off-target Cas9-mediated DNA double-strand breaks, but multiple clones and cell lines were used, and rescue experiments were performed, to maximize reliability (data partly not shown). Moreover, we noted that multiple commercially available antibodies are not specific as evidenced by unaltered staining patterns in confirmed knockout sublines (Supplementary notes). This may explain apparent contradictions between the findings reported here and those of previous studies on CD95L in murine cancer models in vitro and in vivo.

Anyhow, we report here that constitutive CD95 signaling in glioblastoma may be tumor-promoting via both intrinsic growth regulation and immunosuppression. However, CD95 signaling remains complex and the biological context may determine the outcome of modulating this pathway.

## Electronic supplementary material

Below is the link to the electronic supplementary material.

Supplementary Material 1

Supplementary Material 2

Supplementary Material 3

Supplementary Material 4

Supplementary Material 5

Supplementary Material 6

Supplementary Material 7

Supplementary Material 8

Supplementary Material 9

Supplementary Material 10

Supplementary Material 11
