# Supplementary figures and images for: CRISPR/Cas9-mediated abrogation of CD95L/CD95 signaling-induced glioma cell growth and immunosuppression increases survival in murine glioma models
Source: J Neurooncol. 2022 Nov 10;160(2):299–310. doi: 10.1007/s11060-022-04137-x (PMC9722998; doi:10.1007/s11060-022-04137-x)

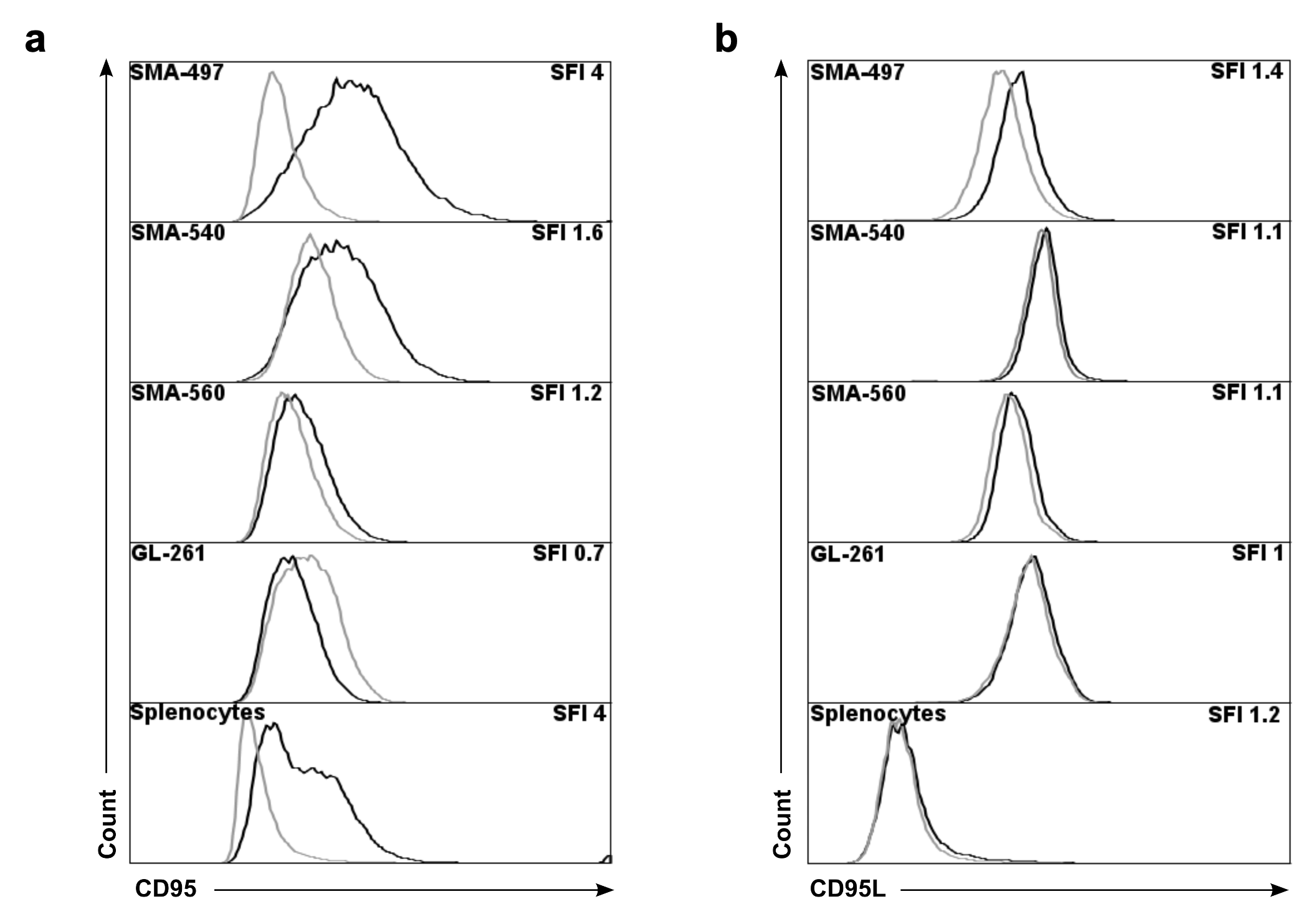

Supplement: Supplementary file 1 — Supplementary Material 1 [file 11060_2022_4137_MOESM1_ESM.png]

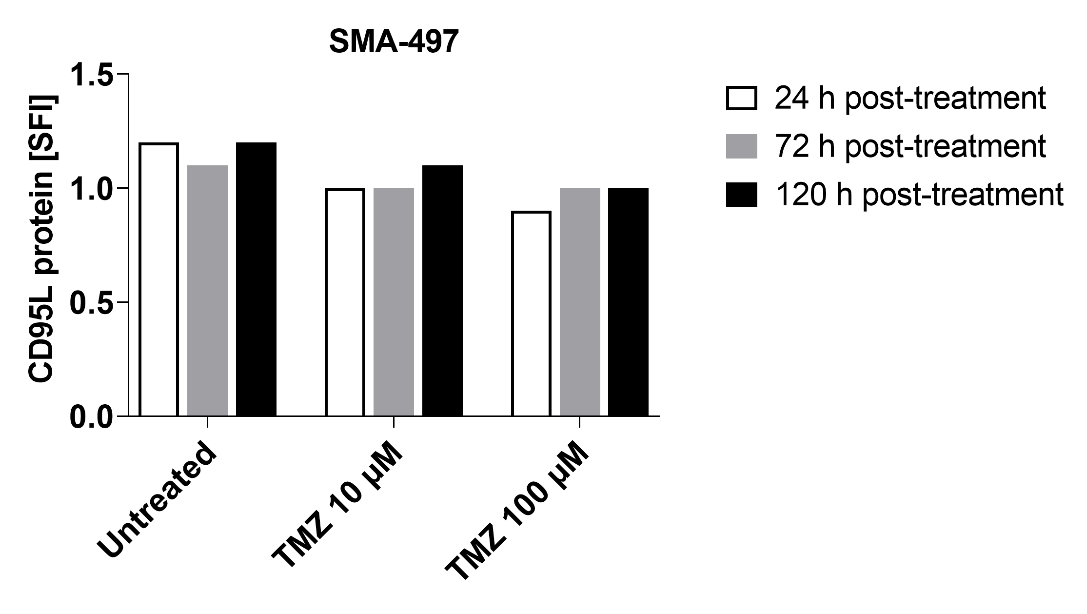

Supplement: Supplementary file 2 — Supplementary Material 2 [file 11060_2022_4137_MOESM2_ESM.png]

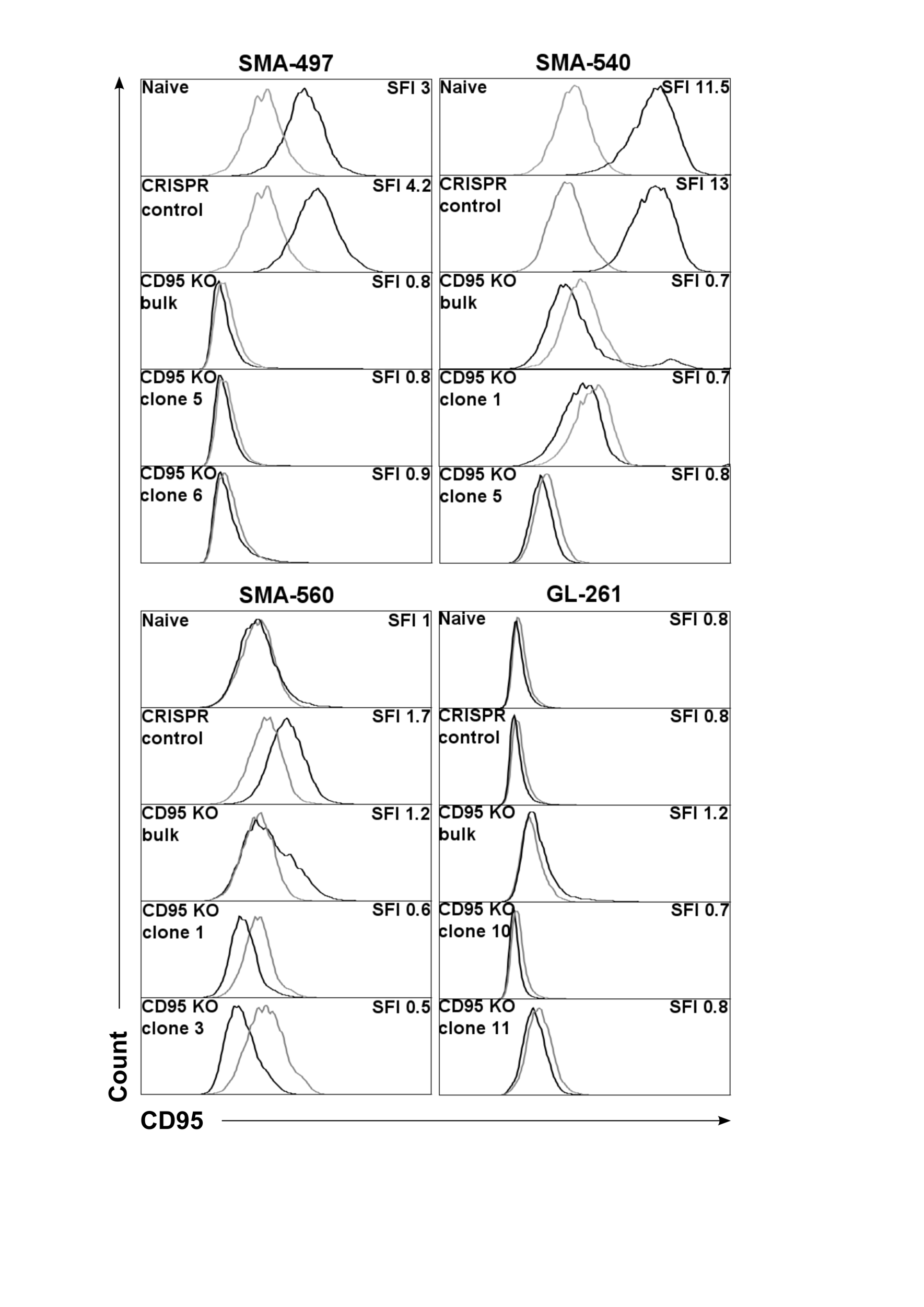

Supplement: Supplementary file 3 — Supplementary Material 3 [file 11060_2022_4137_MOESM3_ESM.png]

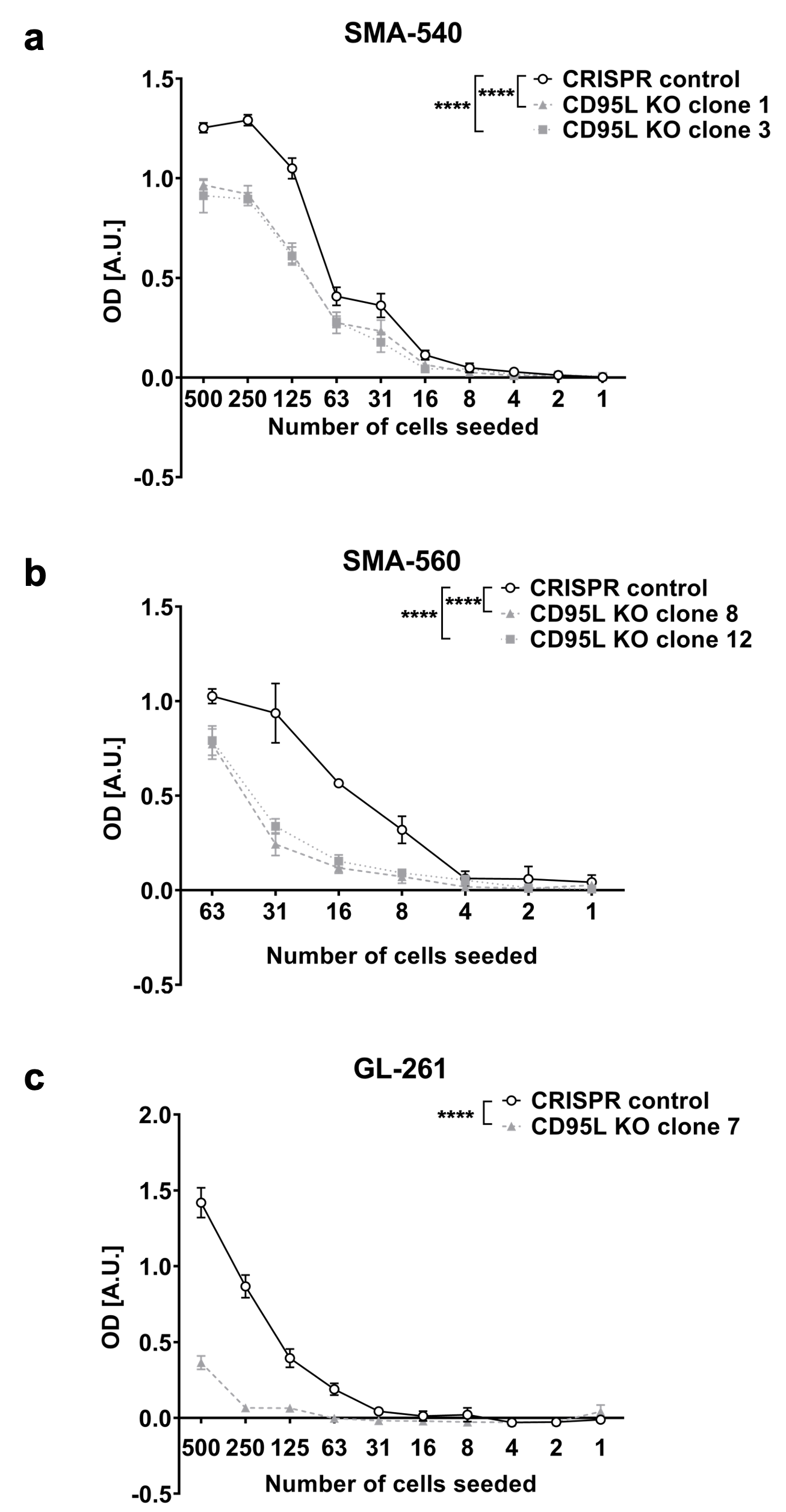

Supplement: Supplementary file 4 — Supplementary Material 4 [file 11060_2022_4137_MOESM4_ESM.png]

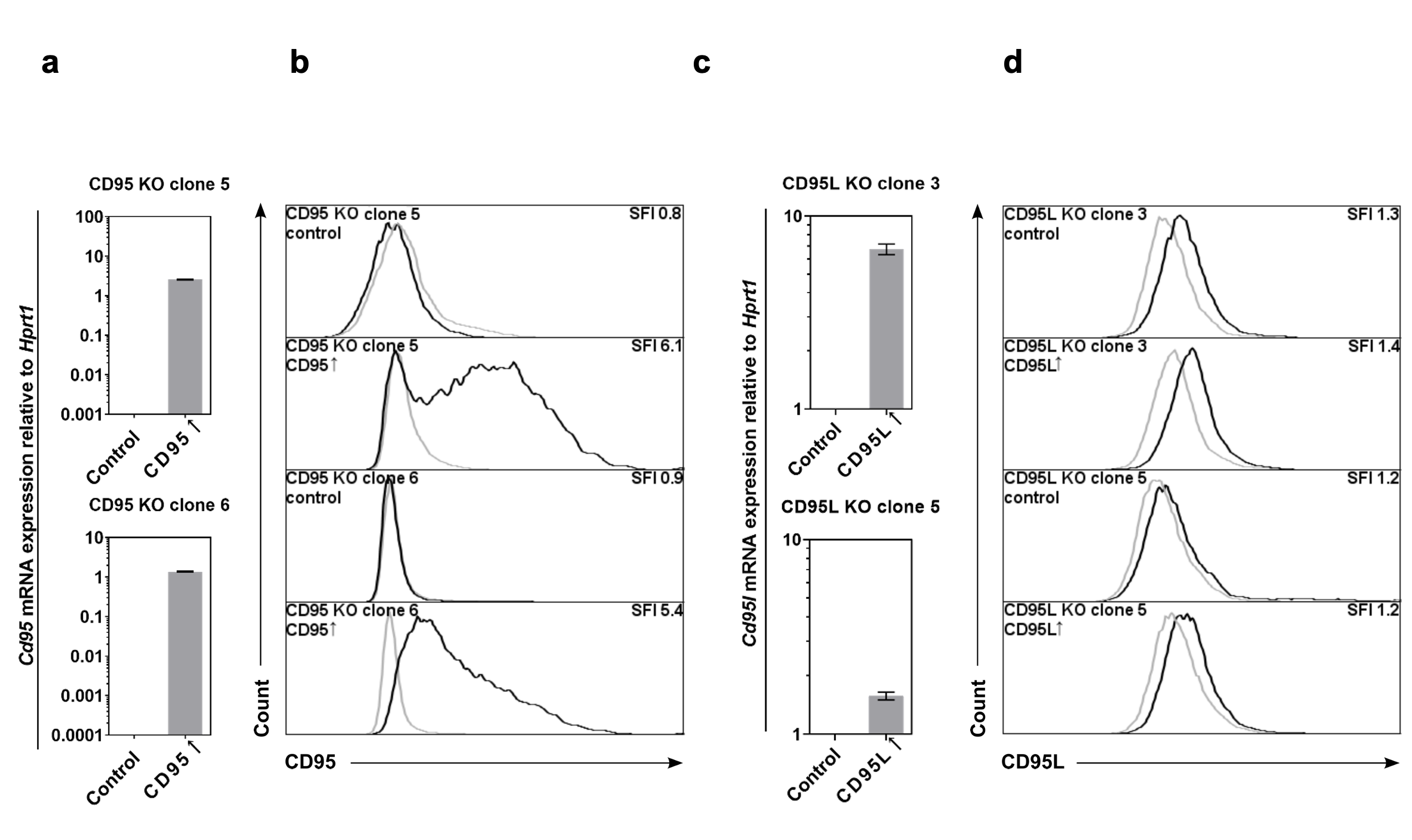

Supplement: Supplementary file 5 — Supplementary Material 5 [file 11060_2022_4137_MOESM5_ESM.png]

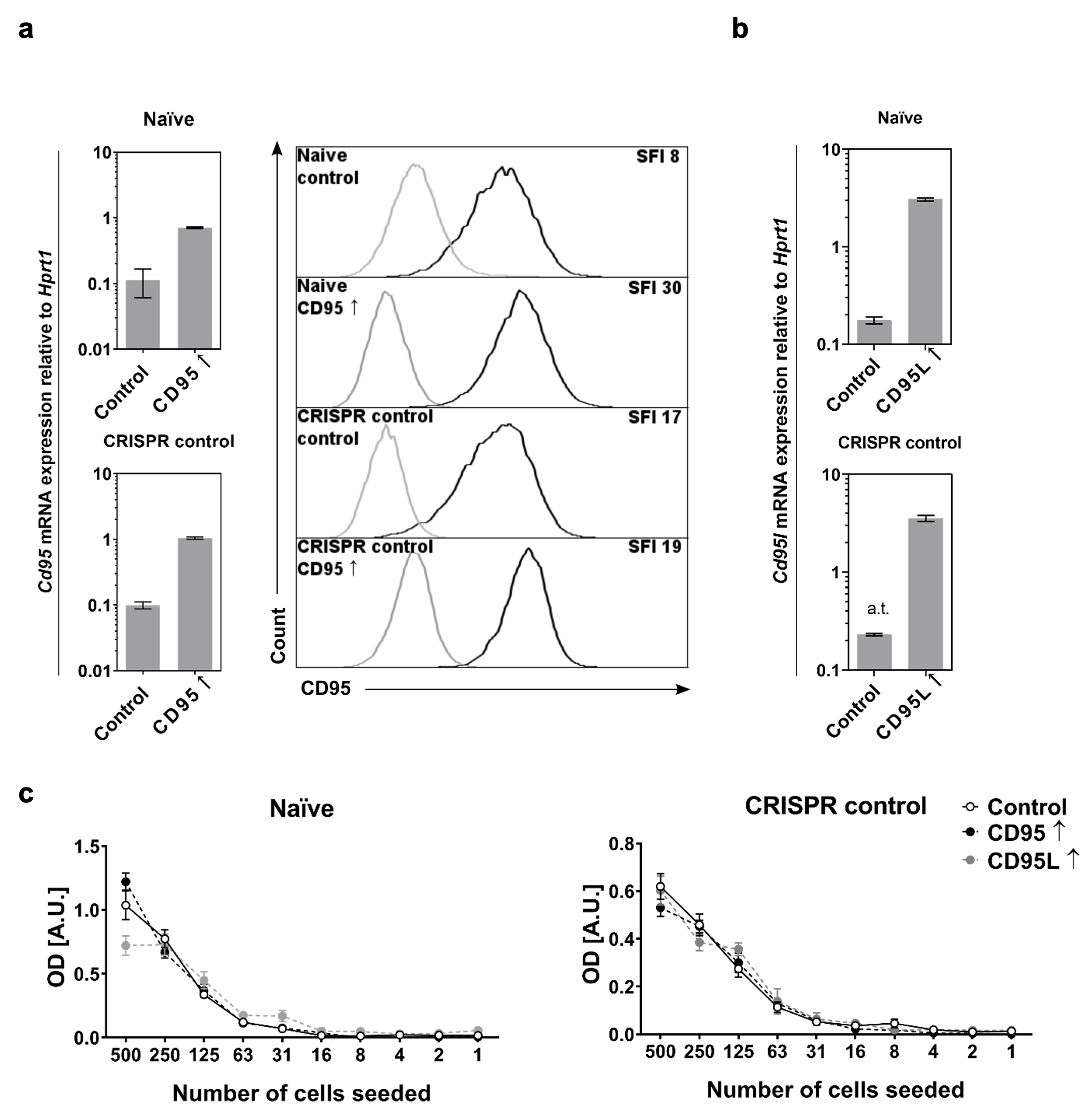

Supplement: Supplementary file 6 — Supplementary Material 6 [file 11060_2022_4137_MOESM6_ESM.png]

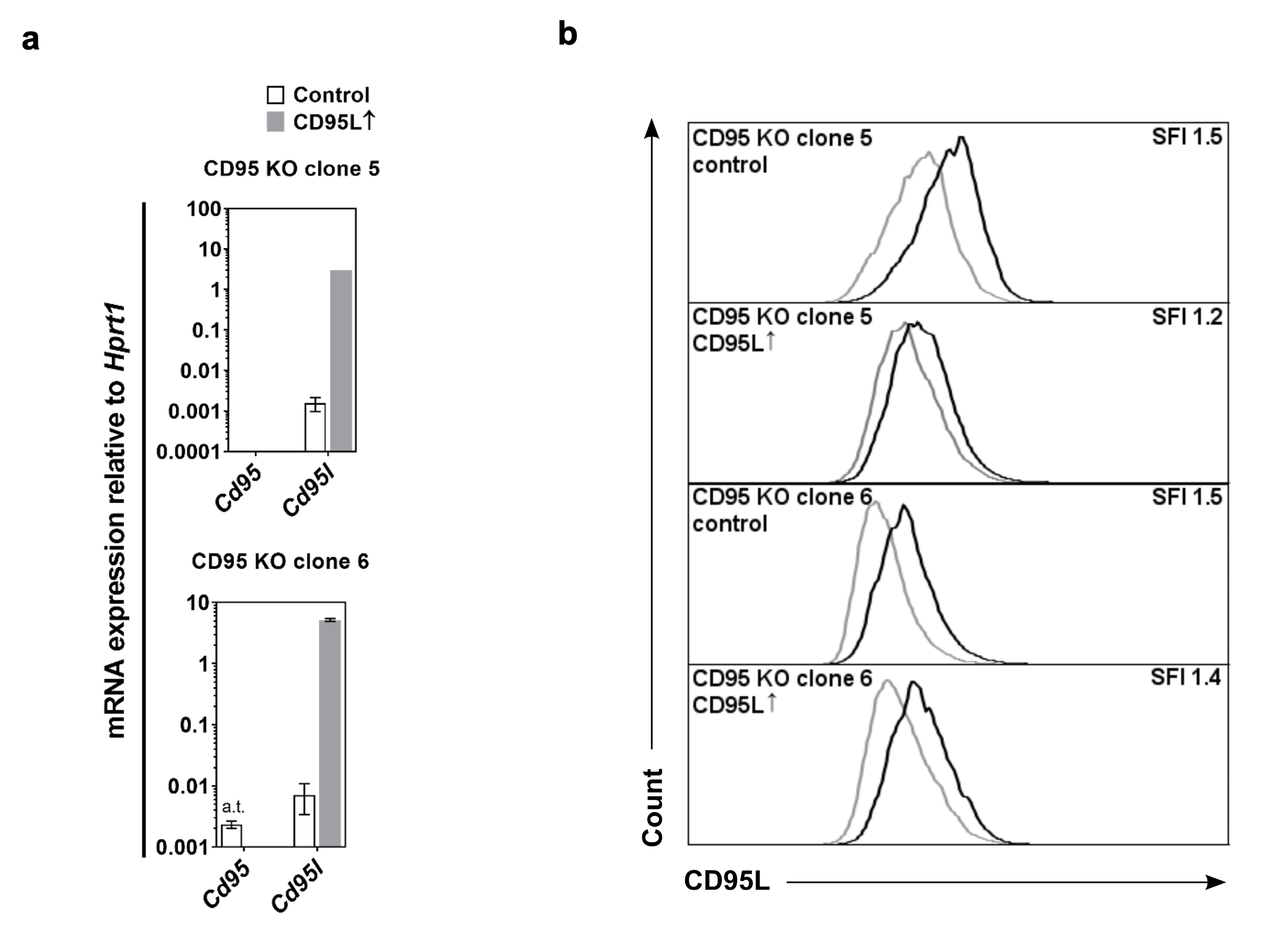

Supplement: Supplementary file 7 — Supplementary Material 7 [file 11060_2022_4137_MOESM7_ESM.png]

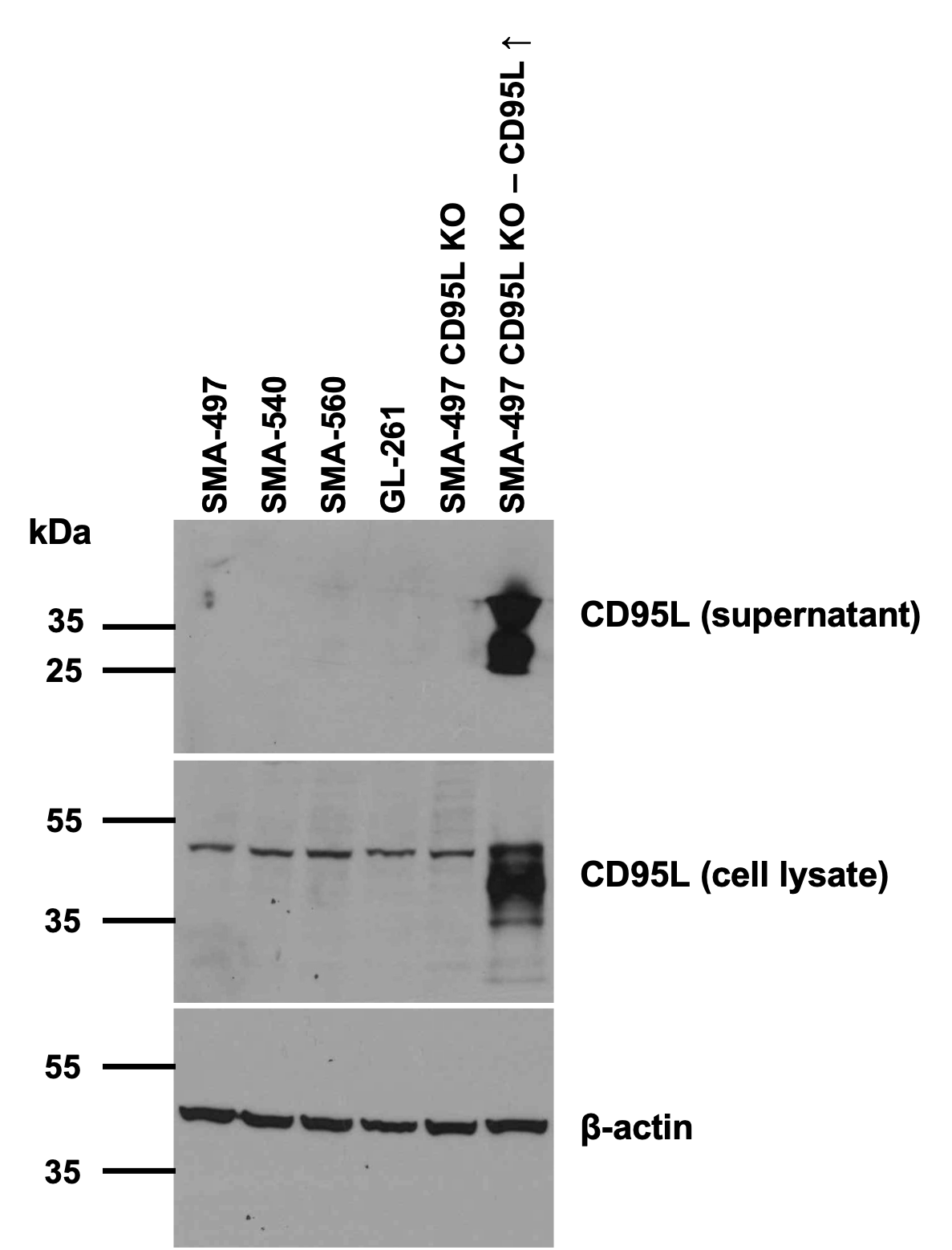

Supplement: Supplementary file 8 — Supplementary Material 8 [file 11060_2022_4137_MOESM8_ESM.png]

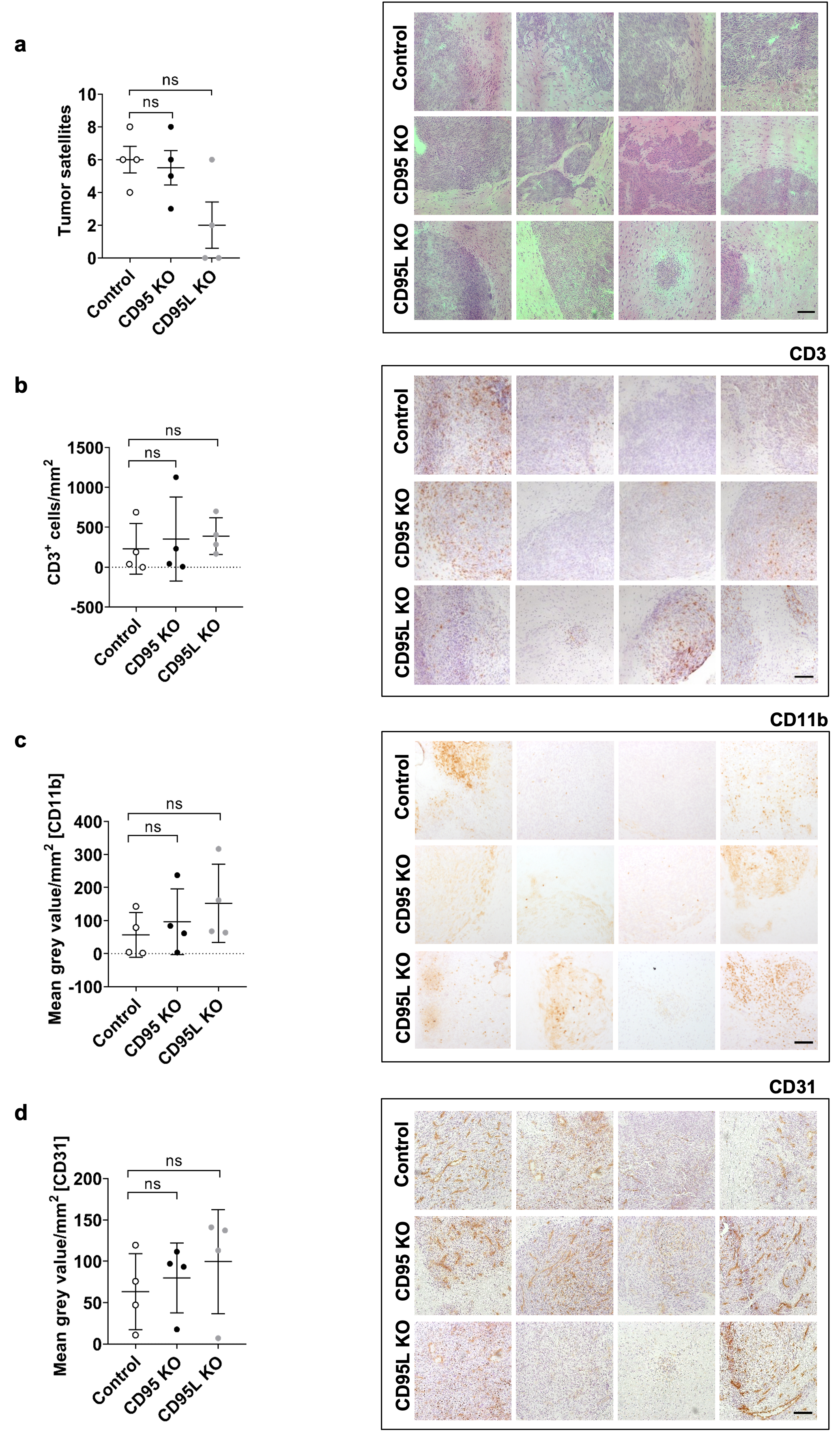

Supplement: Supplementary file 9 — Supplementary Material 9 [file 11060_2022_4137_MOESM9_ESM.png]

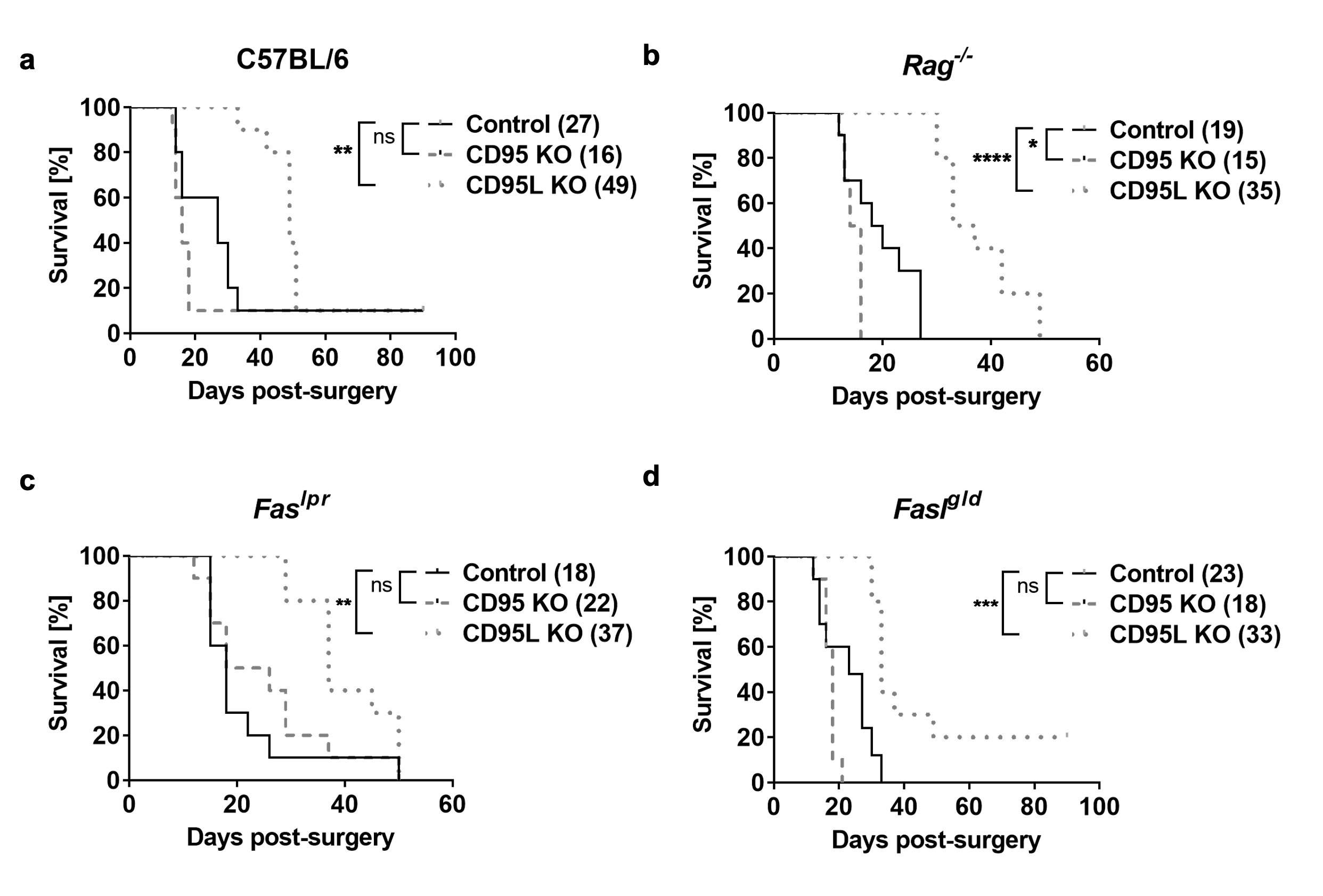

Supplement: Supplementary file 10 — Supplementary Material 10 [file 11060_2022_4137_MOESM10_ESM.png]
