# Supplementary material for: CRISPR/Cas9-mediated abrogation of CD95L/CD95 signaling-induced glioma cell growth and immunosuppression increases survival in murine glioma models
Source: J Neurooncol. 2022 Nov 10;160(2):299–310. doi: 10.1007/s11060-022-04137-x (PMC9722998; doi:10.1007/s11060-022-04137-x)
Supplement: Supplementary file 11 — Supplementary Material 11 [file 11060_2022_4137_MOESM11_ESM.docx]

**Supplementary Material and Methods**

**Reagents**

Mega-Fas-Ligand was kindly provided by TopoTarget S.A. (Lausanne, Switzerland). Cycloheximide was purchased from Santa Cruz Biotechnology (Dallas, TX), staurosporine from VWR (Radnor, PA) and carbobenzoxy-valyl-alanyl-aspartyl-[O-methyl]-fluoromethylketone (zVAD-FMK) from Bachem (Bubendorf, Switzerland). Temozolomide was obtained from Selleckchem (Houston, TX).

***Cd95* and *Cd95l* gene deletion**

*Cd95* or *Cd95l* genes were deleted by means of CRISPR/Cas9 [1]. A pair of single guide RNAs (sgRNA) targeting two different genomic regions of murine *Cd95* or *Cd95l* with minimal predicted off-target binding were designed using the online CRISPR design tool http://tools.genome-engineering.org. The two sgRNA were designed to target coding sequences present in all target gene transcript variants. The sgRNA sequences are indicated below: for *Cd95* deletion 5’-TCTCCGAGAGTTTAAAGCTG-3’, sense and 5’-CTTCATGTGACCTACCAGGT-3’, antisense; for *Cd95l* deletion, 5’- GCTACCGGTGGTATTTTTCA-3’, sense and 5’- AGGTTGAACTACTGCACTAC-3’, sense. sgRNA duplexes were generated by ligation with T4 DNA ligase (Thermo Fisher Scientific, Waltham, MA) at 37ºC/30 min and 95ºC/5 min and cloned into a *pSpCas9*(BB)-2A-*GFP* (PX458) backbone plasmid (#48139, Addgene, Watertown, MA) within BbsI restriction sites. sgRNA insertion in the resulting construct was verified by Sanger sequencing (Microsynth, Balgach, Switzerland). Constructs were transfected by means of the TransIT-X2 delivery system (Mirus Bio, Madison, WI). Ratios of 2/1/60’000 (reagent in ml)/DNA in μg/cell number) were used for SMA-497, SMA-540 and SMA‑560 cells and 5/1/160’000 for GL-261 cells. The selection of transfected cells was carried out by fluorescence-activated cell sorting (FACS) of GFP-positive cells performed two days post-transfection. CD95 and CD95L knockout clonal cell populations were generated upon single cell seeding in 96-well plates. Knockout verification was performed by RT-qPCR using primers spanning the predicted Cas9-mediated double strand DNA break site and flow cytometry. CRISPR control cells were generated by transfection with *pSpCas9*(BB)‑2A‑*GFP* (PX458) plasmids containing each of the following sgRNA: 5’-GCGAGGTATTCGGCTCCGCG-3’ and 5’-ATGTTGCAGTTCGGCTCGAT-3’. These control sgRNA sequences do not target any known sequence in the mouse genome. CRISPR control cells were used as bulk populations.

***Cd95* and *Cd95l* gene delivery**

*Cd95* or *Cd95l* gene transfer was carried out by means of lentivirus-mediated gene delivery. *Cd95* and *Cd95l* sequences were obtained from pBS-(KS) murine *Fas* (cDNA) and pHAXB murine *Cd95l* cDNA (ORF), kindly provided by Pierre Golstein (Marseille, France) and Peter H. Krammer (Heidelberg, Germany), respectively. After a TOPO cloning into pGEM-T Easy vectors (Promega, Madison, WI) intermediate step, cDNA sequences were subjected to PCR-mediated SpeI and EcoRI restriction site generation and amplification using the following primers: for *Cd95*, forward 5’-AAAGTTACTAGTATGCTGTGGATCTGGGTC-3’, reverse 5’-AAGGACAATGTCTGGAGTGAGAATTCAAAGTT-3’; for *Cd95l* forward 5’-ATTGTTACTAGTATGCAGCAGCCCATG-3’, reverse 5’-TTTCGGCTTGTATAAGCTTTAAGAATTCATTGTT-3’.

Amplicons were subcloned, within SpeI and EcoRI restriction sites, into a lentiviral transfer vector (#39481, Addgene) under a human cytomegalovirus promoter [2]. Insert presence and integrity in the lentiviral constructs was verified by Sanger sequencing (Microsynth). For lentivirus generation, HEK 293T cells were transfected with the above-mentioned construct, the VSV-G envelope-expressing plasmid pMD2.G (plasmid #12259, Addgene) and the packaging plasmid pCMV-dR8.91 (#2221, Addgene) by means of CaPO_4_ precipitation. Glioma cells were transduced with PEG-*it* (System Biosciences, Palo Alto, CA)-concentrated lentivirus particles using 8 μg/ml polybrene (Santa Cruz Biotechnology). Transduced cells were selected with 6 μg/ml puromycin (Thermo Fisher Scientific) for 10 days. CD95 or CD95L expression was assessed by RT-qPCR, flow cytometry or immunoblot.

**RT-qPCR**

For gene expression analyses, cells were deprived of serum for 24 h. Total mRNA was isolated by means of the NucleoSpin®RNA II kit (Macherey-Nagel, Dueren, Germany) and cDNA was synthesized by means of the High Capacity cDNA Reverse Transcription kit (Thermo Fisher Scientific). Fifteen ng cDNA were amplified with the PowerUp SYBR Green Master Mix in a QuantStudio 6 Real-Time PCR System (Thermo Fisher Scientific). Thermal cycling was carried out as follows: 50ºC/2 min, 95ºC/2 min and 40 cycles at 95ºC/15 sec and 60ºC/1 min. cDNA amplification was computed by the QuantStudio software V1.2 (Thermo Fisher Scientific) and relative quantification of transcript expression was calculated using the primer efficiency‑weighted comparative C_T_ (∆C_T_) method. Specific transcript expression was normalized to hypoxanthine-guanine phosphoribosyltransferase 1 (*Hprt1*) as a housekeeping gene for gene expression analysis in glioblastoma cells (Valente et al., 2009). To ensure reliable quantification, only C_T_ values of at most 32 cycles for the target loci were considered indicative of gene expression. C_T_ values above this threshold failed to show reproducibility or specificity. The primers used (100 nM) are listed in Table S1.

**Flow cytometry**

Flow cytometry assays were performed with cells subjected to serum deprivation for 24 h. Cells were dissociated with Accutase (Thermo Fisher Scientific) and stained with adequate antibodies and viability dye. For splenocyte and tumor tissue protein staining, incubation with adequate antibodies was preceded by a 15 min incubation at 4ºC with a CD16/CD32 Fc receptor blocking reagent (#553142 1:100; BD Biosciences). The following antibodies were used at 4ºC for 30 min: Alexa Fluor 647 hamster anti-mouse CD95 clone Jo2 (#563647 1:100), PE hamster anti-mouse CD95L clone MFL3 (#555293 1:50), all from BD Biosciences. Parallel aliquots of cells were stained with matching isotype controls. Zombie Aqua and Zombie NIR fixable viability kits (BioLegend) were used as viability dyes for live/dead staining. Data acquisition was performed with a BD FACSVerse flow cytometer (BD Biosciences). Data analysis was performed with FlowJo (Tree Starm Stanford, CA). Only specific fluorescence indexes (SFI) higher than 1.5 were considered indicative of protein detection.

**Fluorescence-activated cell sorting (FACS)**

Accutase (Thermo Fisher Scientific)-dissociated single cell suspensions were filtered through a 35 μm cell strainer (Corning, NY). Cell sorting was performed with a 100 μm or a 130 μm nozzle in a FACSAria III cell sorter under sterile conditions. Sorted cells collection was carried out in culture medium for subsequent cell expansion and subline generation.

**Immunoblot**

Cell lysates for the analysis of intracellular and transmembrane proteins were obtained from cells subjected to serum deprivation for 24 h. Cells were lysed with RIPA lysis buffer (Millipore, Burlington, MA) supplemented with 100 μg/mL phenylmethylsulfonyl fluoride and protease and phosphatase inhibitor cocktails (Sigma-Aldrich). Cell supernatants for the analysis of soluble proteins were collected upon a 24 h serum starvation period when applicable and concentrated using 3’000 nominal molecular weight limit Amicon centrifugal filter devices (Millipore). Protein was quantified by Pierce bicinchoninic assay (Thermo Fisher Scientific). Uniform protein amounts (30 μg lysate protein/lane or 80 μg supernatant protein/lane) were loaded into 10% acrylamide/bis gels. Protein separation was performed by SDS-PAGE under reducing conditions and proteins were transferred to 0.45 μM-pore nitrocellulose membranes (BioRad, Hercules, CA). To prevent non-specific antibody binding, membranes were blocked in 5% skim milk in Tris-buffered saline buffer containing 0.1% Tween 20. Thereafter, membranes were incubated at 4ºC for 16 h with a rat anti-mouse CD95L clone 101626 (#5262 1:100) primary antibody from R&D Systems (Minneapolis, MN). Membranes were subsequently incubated for 1 h at room temperature with a goat anti-rat IgG1 (#2065 1:5000) HRP-conjugated secondary antibody from Santa Cruz Biotechnology. Detection of β-actin was directly achieved by incubation with a HRP-conjugated anti-β-actin antibody (#47778 1:5000) from Santa Cruz Biotechnology for 1 h at room temperature. For chemiluminescent protein detection, membranes were incubated with Pierce ECL immunoblot substrate and exposed to x-ray films for subsequent development.

**Apoptosis assays**

Apoptotic cell death was inferred from caspase 3/7-like N-acetyl Asp‑Glu‑Val‑Asp‑7‑amino-4-methylcoumarin (Ac-DEVD-amc) cleaving activity. Ten thousand cells per well were seeded in 96-well plates and treated for 6 h with Mega‑Fas-Ligand, cycloheximide, staurosporine, or zVAD-fmk under serum-free conditions. Cells were lysed with lysis buffer P containing 25 mM Tris-HCl pH 8, 120 mM NaCl, 5 mM EDTA and 0.5% NP-40 and exposed to 6.25 µM Ac-DEVD-amc. Fluorometric reading of Ac-DEVD-amc cleavage was performed at 380ex/450em in an Infinite M200 PRO plate reader (Tecan, Maennedorf, Switzerland).

**Cell growth assessment in limiting dilution assays**

Cells were seeded at decreasing cell densities of 500 to 1 cells/well in 96-well plates and incubated for 7 days. To evaluate end-point cell growth, cells were stained with 0.5% crystal violet (Sigma-Aldrich).

**Flow cytometry-based T cell lysis assays**

Mouse splenocytes were isolated from C57BL/6 or VM/Dk mice spleens. Tissue was mechanically dissociated and subjected to red blood cell lysis with ACK buffer (17 mM Tris–HCl (pH 7.2), 144 mM NH4Cl). Splenocytes were seeded at a density of 10^6^ cells/ml in advanced Roswell Park memorial Institute (RPMI) 1640 medium supplemented with 10% FCS, 2 mM L-glutamine (Thermo Fisher Scientific), 1% penicillin/streptomycin (Sigma-Aldrich) and 55 μM 2-mercaptoethanol (Gibco). Splenocyte activation was induced with 2 μg/ml concanavalin A (Sigma-Aldrich) for 20 h. Thereafter, splenocytes were cultured in 25 IU/ml recombinant IL-2 (Peprotech)-containing medium. Activated splenocytes were labelled with the PKH26 Red Fluorescent Cell Linker Kit for general cell membrane staining (Sigma-Aldrich) and co-cultured with SMA-497, SMA-540, SMA-560 or GL-261 in a 1:1 ratio for 24 h. Cancer cell-mediated splenocyte lysis was assessed by flow cytometry. Lysed splenocytes were identified based on positive PKH26 and positive Zombie-NIR (BioLegend) staining. Alternatively, unstained splenocytes were co-cultured with murine glioma cells and gated based on positive CD3 and positive Zombie NIR staining. CD3 staining was achieved with a PerCP-Cy5.5-conjugated anti-mouse CD3, clone 17A2 antibody (1:50, BioLegend). The percentage of lysed splenocytes was calculated by subtracting the proportion of dead splenocytes in monoculture [5].

**Animal studies**

Crl:CD1-*Foxn1^nu^*, *Fas^lpr^*/J and *Fasl^gld^*/J mice were purchased from Charles River Laboratories (Sulzfeld, Germany), C57BL/6NRj mice were purchased from Janvier Labs (Le Genest-Saint-Isle, France) and VM/Dk and C57BL/6 *Rag^-/-^* mice were bred in pathogen-free facilities at the University of Zurich. Mice of 4 to 16 weeks of age were used in all experiments. Mice were implanted with 5’000-100’000 SMA-497 cells or 500 GL-261 cells. Orthotopic xenograft and syngeneic gliomas were generated by means of stereotactic surgical injection of glioma cells with a 26s gauge syringe (Hamilton, Reno, NV). Glioma cells were implanted into the right mouse striatum to a 3 mm depth, 2 mm lateral and 1 mm posterior to the bregma. For gene knockout comparison experiments, each experimental group consisted of equal numbers of mice implanted with each of the two sublines with the same genotype (i.e., naïve and CRISPR control cells, two CD95 knockout clones or two CD95L knockout clones). Mice were regularly controlled for neurological symptoms. The onset of neurological symptoms defined end-stage survival. Unless stated otherwise, end-stage survival was assessed in 7-10 mice per group and 4 pre-randomized mice per group were euthanized at the onset of neurologic symptoms in the first experimental mouse for histological analyses.

**Histology and immunohistochemistry**

Mouse brains were cryopreserved with cryochrome embedding resin (Thermo Fisher Scientific). Brains were cut in 8 μm thick sections with a Microm HM 560 cryostat (Thermo Fisher Scientific). Every 20^th^ tumor section was stained with hematoxylin and eosin (H&E) for tumor volumetry and satellite number evaluation. Tumor volumetry was performed using an ellipsoid geometric primitive approximation [6, 7]. Tumor satellites were quantified based on their definition as tumor cell aggregates of at least 10 cells located 3 cell layers distant from the main tumor mass [8]. For immunohistochemical stainings, cryosections were fixed with 4% formaldehyde and endogenous peroxidase was inactivated with 3% H_2_O_2._ Blocking with Super Block (ScyTek Laboratories, West Logan, UT) was followed by a 16 h incubation at 4ºC with the following primary antibodies: rat anti-mouse CD3 clone 17A2 (#555273 1:100), rat anti-mouse CD11b clone M1/70 (#550282 1:500), rat anti-mouse CD31 (#550274 1:50), all from BD Biosciences. Cryosections were stained in parallel with matching rat IgG2b κ and rat IgG2a isotype controls. After primary staining, cryosections were incubated for 20 min at room temperature with the anti-rat immune-peroxidase polymer Histofine Simple Stain MAX PO (R) (Nichirei). Thereafter, cryosections were exposed to ImmPACT DAB peroxidase substrate (Vector Laboratories), counterstained with hematoxylin, dehydrated and mounted on coverslips using Rotimount mounting medium. Image acquisition was performed with an AxioCam MRc camera coupled to an Axio Scope.A1 microscope and processed with the AxioVision LE64 program. Immunohistochemical staining quantification was obtained from four tumor regions, covering tumor edges and center and performed with ImageJ software. The number of CD3^+^ cells was calculated per tumor mm^2^. CD31 and CD11b levels were assessed semi‑quantitatively by measuring mean grey values within the tumor area upon color deconvolution [9]. Immunohistochemical stainings were likewise performed with the anti-CD95 antibody #82419 (Abcam, Cambridge, UK) and the anti-CD95L antibodies APG1181 (kindly provided by Apogenix AG, Heidelberg, Germany) or #15285 (Abcam).

**Supplementary Notes**

**Note S1**

Immunoblot and immunocytochemical stainings with the anti‑CD95 antibodies #82419 (Abcam) and #715 (Santa Cruz Biotechnology) resulted in suboptimal detection or specificity (data not shown). Immunoblot was performed as described in the Supplementary Material and Methods section. For immunocytochemical stainings, cytospin monolayers of serum-deprived (24 h) cells were prepared using a Cytospin 4 centrifuge (Thermo Fisher Scientific). Cytospins were fixed with 4% formaldehyde. Potential endogenous peroxidase activity was inhibited with 3% H_2_O_2_. Blocking with a serum-free protein blocking reagent (Super Block, ScyTek Laboratories, Logan, UT) was followed by primary antibody incubation at 4ºC for 16 h. Cytospins were thereafter incubated at room temperature for 20 min with the anti-rabbit immune‑peroxidase polymer Histofine Simple Stain MAX PO (R) (Nichirei, Tokyo, Japan), exposed to ImmPACT DAB peroxidase substrate (Vector Laboratories, Burlingame, CA) and counterstained with hematoxylin. Thereafter, cytospins were dehydrated twice in 96% ethanol, 100% ethanol and four times in Roticlear (Carl Roth, Karlsruhe, Germany). None of the above-mentioned antibodies resulted in specific staining.

**Note S2**

CD95Ls protein expression was investigated by means of intracellular flow cytometry staining with the anti-CD95L clone MFL3 (#555293, BD Biosciences) and immunoblot with the monoclonal anti-CD95L antibody #MAB5262 (R&D systems), both of which recognize part of the extracellular domain present in CD95Ls. CD95Ls protein was not detected by either means (data not shown), indicating undetectability of CD95L levels or lack of antibody affinity for CD95Ls, since the immunogens used for the generation of both antibodies were derived from full-length CD95L. Antibodies specifically detecting CD95Ls are lacking.

**Note S3**

CD95L protein detection by immunoblot and immunocytochemistry was attempted with the anti-CD95L antibodies #15285 (Abcam) and #834 (Santa Cruz Biotechnology) which are supposed to detect the CD95L cytoplasmic domain. However, data could not be interpreted due to lack of specificity. Specifically, this was demonstrated by persistence of the signal in cell lines depleted of CD95L mRNA.

**Note S4**

Tumoral CD95 and CD95L levels could not be assessed since the immunohistochemical evaluation with none of the antibodies tested (anti-CD95 #82419 from Abcam and anti-CD95L APG1181 from Apogenix AG or #15285 from Abcam) resulted in specific staining on brain cryosections.

**Note S5**

**Tumor intrinsic effects only mediate increased survival upon CD95L knockout in the GL-261 model.**

C57BL/6 mice implanted with CD95L knockout GL‑261 cells showed increased survival compared to mice implanted with control GL-261 cells, but few long-term survivors were observed (Fig. S10). CD95 knockout did not confer a survival benefit, but GL-261 cells express very low levels of CD95 in vitro (Fig. 1) and do not induce CD95 in vivo as assessed by examining GFP-labeled GL-261 mice in the brains of syngeneic mice (data not shown). Survival upon implantation of CD95 or CD95L knockout GL-261 cells was additionally compared between wildtype mice and T and B cell-deficient *Rag^-/-^* mice. The improved survival upon CD95L knockout observed in immunocompetent C57BL/6 mice was also seen in *Rag^-/-^* mice (Fig. S10a,b), indicating that the survival advantage of mice inoculated with CD95L knockout GL-261 cells was tumor-intrinsic rather than immune-dependent. Accordingly, we also explored whether, in addition to CD95 or CD95L expressed by the glioma cells, CD95 or CD95L expressed by the host might influence survival of glioma-bearing mice, but the survival gain afforded by *Cd95l* gene deletion was maintained in mice lacking functional CD95 (*Fas^lpr^*) or functional CD95L (*Fasl^gld^*) (Fig. S10c,d).

**Supplementary Table**

**Table S1. List of RT-qPCR primers.**

| ***Cd95* transcript expression assessment and *Cd95l* transcript variant identification** | |
| --- | --- |
| *Cd95* | forward 5’-CATCTCCGAGAGTTTAAAGCTG-3’ |
|  | reverse 5’-CCTCAACTTTTTTTTTACCAGGTTG-3’ |
| *Cd95l* exon 1 | forward 5’-GGCTACCGGTGGTATTTTTCA -3’ |
|  | reverse 5’-GTTGGTGAACTCACGGAGTT-3’ |
| *Cd95l* exon 3-4 | forward 5’-CAGGGAACCCCCACTCA-3’ |
|  | reverse 5’-GCCCATATCTGTCCAGTAGT-3’ |
| *Cd95l* exon 4 | forward 5’-GAGGAACTCTAAGTATCCTGAGG-3’ |
|  | reverse 5’-GCCCATATCTGTCCAGTAGT-3’ |
| **Internal control gene expression quantification** | |
| *Hprt1* | forward 5’-CCTAAGATGAGCGCAAGTTGAA-3’ |
|  | reverse 5’-CCACAGGACTAGAACACCTGCTAA-3’ |

Of these primers, the following were used for gene deletion verification upon CRISPR/Cas9-mediated knockout: *Cd95*, which spans the predicted Cas9 dsDNA break sites guided by both *Cd95* sgRNA; *Cd95l* exon 1, which overlaps the predicted Cas9 dsDNA break sites guided by *Cd95l* sgRNA 1; *Cd95l* exon 4, which overlaps the predicted Cas9 dsDNA break sites guided by *Cd95l* sgRNA 2.

**Supplementary Figure Legends**

**Fig. S1. CD95 and CD95L expression in mouse glioma cells – additional data.** a,b. Flow cytometry histograms illustrating CD95 and CD95L cell surface protein levels in SMA-497, SMA-540, SMA-560 and GL-261 cells (corresponding to the data in Fig. 1b,d) are depicted. Specific fluorescence indexes (SFI) indicated in the right upper corner were calculated dividing the median fluorescence intensities of the experimental antibody (black) and the isotype control (grey).

Fig. S2. CD95L expression in mouse glioma cells is not induced by temozolomide (TMZ). SMA‑497 cells were untreated or treated with 10 µM or 100 µM temozolomide for 24 h and cell surface CD95L protein levels were quantified by flow cytometry (SFI, specific fluorescence index calculated dividing the median fluorescence intensities of the anti-CD95L antibody and the isotype control).

**Fig. S3.** **CD95 protein levels in CD95 knockout (KO) SMA-497, SMA-540, SMA-560 and GL-261 mouse glioma cells – additional data.** Flow cytometry histograms corresponding to the grey columns (right y axis) in Fig. 3a are depicted. Bulk KO cells, from which CD95 KO clonal sublines were derived, are depicted for comparison. Specific fluorescence indexes (SFI) were calculated dividing the median fluorescence intensities of the anti-CD95 antibody (black) and the corresponding isotype control (grey).

Fig. S4. Effect of CD95L knockout (KO) on SMA-540, SMA-560 and GL-261 cell growth in vitro. a-c. Growth of CRISPR control or CD95L KO SMA-540 (a), SMA-560 (b) or GL-261 (c) cells was estimated by crystal violet staining in limiting dilution assays of cells grown in adherence conditions in the presence of fetal calf serum. Data are expressed as mean and SEM of a representative experiment. Data were reproduced in three independent experiments. Main column effect statistical significances were determined by means of a two-way ANOVA test followed by Bonferroni’s post hoc test. A.U., arbitrary units; ns, not significant; ***, p<0.001; ****, p<0.0001.

Fig. S5. Transfection of *Cd95* or *Cd95l* in CD95 knockout (KO) or CD95L KO cells. CD95 KO or CD95L KO SMA-497 clonal sublines were transfected with CD95 or CD95L by means of lentiviral gene delivery. a,c. Expression of *Cd95* and *Cd95l* mRNA was assessed by RT-qPCR using *Hprt1* as internal control. *Cd95l* expression was assessed using primers targeting both transcript variants. b,d. CD95 and CD95L cell surface protein expression was assessed by flow cytometry. SFI, specific fluorescence index (calculated experimental antibody, black, median fluorescence intensity ÷ isotype control, grey, median fluorescence intensity).

**F**ig. S6. Overexpression of CD95 or CD95L in naïve and CRISPR control cells. SMA-497 cells were transfected with *Cd95* or *Cd95l* by lentivirus-mediated gene delivery. a,b. Expression of *Cd95* and both *Cd95l* transcripts was assessed by RT-qPCR using *Hprt1* as internal control and cell surface CD95 levels were assessed by flow cytometry. c. Growth of naïve or CRISPR control cells engineered to overexpress CD95 or CD95L was assessed by crystal violet staining in limiting dilution assays. Data in a,b are expressed as mean and SD. Data in c are expressed as mean and SEM of representative experiments. Data in c were compared by means of a two-way ANOVA test followed by Bonferroni’s post hoc test (main column effect). Statistical significances were not revealed. Data were reproduced in three independent experiments. a.t., above reliability C_T_ threshold (C_T_>32); SFI, specific fluorescence index (calculated dividing the mean fluorescence intensity of the anti-human CD95 clone DX2 antibody, black, and the isotype control, grey, mean fluorescence intensity); A.U., arbitrary units. Naïve refers to non-transfected cells.

**F**ig. S7. Overexpression of CD95L in CD95 knockout (KO) cells. CD95 KO SMA-497 cells were transfected with *Cd95l* by means of lentivirus-mediated gene delivery. a,b. Expression of *Cd95* and *Cd95l* (using primers targeting both known *Cd95l* transcript variants) mRNA in CD95 KO clone 5 and CD95 KO clone 6 was assessed by RT-qPCR using *Hprt1* as internal control (a) and cell surface protein levels were assessed by flow cytometry (b). a.t., C_T_ values above reliability threshold (32).

**F**ig. S8. Additional evaluation of expression of CD95L in *Cd95l* transfectants. CD95L protein detection by immunoblot in cell lysates and cell supernatants of naïve SMA-497, SMA‑540, SMA-560 and GL-261 cells, CD95L knockout, KO, (clone 3) SMA-497 cells and *Cd95l*‑transfected CD95L KO (clone 3) SMA-497 cells. β-actin was used as a loading control of cell lysate protein. Supernatant loading was controlled by means of Ponceau staining.

**Fi**g. S9. Phenotypic analysis of CD95 and CD95L knockout (KO) in syngeneic mouse SMA-497 gliomas. Syngeneic VM/Dk and athymic *Foxn1^nu^* mice were orthotopically implanted with control (naïve or CRISPR control), CD95 KO (clones 5 and 6) or CD95L KO (clones 3 and 5) SMA-497 cells and brain sections from animals sacrificed when the first mouse became symptomatic were studied. a. Tumor satellites consisting of more than 10 cells separated by three cell layers from the main tumor mass were quantified. b,c. Immune cell infiltration was evaluated by means of CD3 (b) and CD11b (c) staining. d. Vessel density was assessed based on CD31 staining. Representative images of each individual brain section included in the analyses are shown. Data are expressed as mean and SD of n = 4. Statistical significances between CD95 KO or CD95L KO cells and control cells were determined by means of a one‑way ANOVA test followed by Bonferroni’s post hoc test. n.s., not significant. Scale bar = 100 μm.

Fig. S10. Effect of CD95 and CD95L knockout (KO) and functional CD95 and CD95L deficiency in host in the GL-261 mouse glioma model**.** Wildtype C57BL/6 (a), *Rag*^-/-^ (b), functional CD95-deficient (*Fas^lpr^*) (c) or CD95L-deficient (*Fasl^gld^)* (d) mice were orthotopically implanted with control (naïve and CRISPR control), CD95 KO (clones 10 and 11) or CD95L KO (clone 7) GL-261 cells. Kaplan-Meier survival curves depicting end-stage survival are shown. Median survival in days is shown in brackets (n = 10 mice per group). Statistical significances between CD95 KO or CD95L KO and control glioma-bearing mice within the same mouse strain were determined by log-rank test. ns, not significant; *, p<0.05; **, p>0.01; ***, p<0.001; ****, p<0.0001.

**Supplementary References**

1. Ran FA, Hsu PD, Wright J, et al (2013) Genome engineering using the CRISPR‑Cas9 system. Nature Protocols 8:2281–2308. https://doi.org/10.1038/nprot.2013.143

2. Guan B, Wang TL, Shih IM (2011) ARID1A, a factor that promotes formation of SWI/SNF-mediated chromatin remodeling, is a tumor suppressor in gynecologic cancers. Cancer Research 71:6718–6727. https://doi.org/10.1158/0008-5472.CAN-11-1562

3. von dem Knesebeck A, Felsberg J, Waha A, et al (2012) RANK (TNFRSF11A) Is Epigenetically Inactivated and Induces Apoptosis in Gliomas. Neoplasia 14:526–534. https://doi.org/10.1596/neo.12360

4. Valente V, Teixeira SA, Neder L, et al (2009) Selection of suitable housekeeping genes for expression analysis in glioblastoma using quantitative RT-PCR. BMC Molecular Biology 10:62–63. https://doi.org/10.1186/1471-2199-10-17

5. Weiss T, Schneider H, Silginer M, et al (2018) NKG2D-Dependent antitumor effects of chemotherapy and radiotherapy against glioblastoma. Clinical Cancer Research 24:882–895. https://doi.org/10.1158/1078-0432.CCR-17-1766

6. Schmidt KF, Ziu M, Ole Schmidt N, et al (2004) Volume reconstruction techniques improve the correlation between histological and in vivo tumor volume measurements in mouse models of human gliomas. Journal of Neuro-Oncology 68:207–215. https://doi.org/10.1023/B:NEON.0000033364.43142.bf

7. Schneider H, Lohmann B, Wirsching HG, et al (2017) Age-associated and therapy-induced alterations in the cellular microenvironment of experimental gliomas. Oncotarget 8:87124–87135. https://doi.org/10.18632/oncotarget.19894

8. Seystahl K, Papachristodoulou A, Burghardt I, et al (2017) Biological role and therapeutic targeting of TGF-b3 in glioblastoma. Molecular Cancer Therapeutics 16:1177–1186. https://doi.org/10.1158/1535-7163.MCT-16-0465

9. Crowe A, Yue W (2019) Semi-quantitative Determination of Protein Expression Using Immunohistochemistry Staining and Analysis: An Integrated Protocol. Bio-Protocol 9:e3465. https://doi.org/10.21769/bioprotoc.3465
